# Autonomous adaptive optimization of NMR experimental conditions for precise inference of minor conformational states of proteins based on chemical exchange saturation transfer

**DOI:** 10.1371/journal.pone.0321692

**Published:** 2025-05-16

**Authors:** Takuma Kasai, Takanori Kigawa

**Affiliations:** 1 Laboratory for Cellular Structural Biology, RIKEN Center for Biosystems Dynamics Research, Yokohama, Kanagawa, Japan; 2 Research DX Foundation Team, TRIP Headquarters, RIKEN, Yokohama, Kanagawa, Japan; 3 NMR Operation Team, RIKEN Center for Biosystems Dynamics Research, Yokohama, Kanagawa, Japan; Zhejiang University College of Life Sciences, CHINA

## Abstract

In scientific experiments where measurement sensitivity is a major limiting factor, the optimization of experimental conditions, such as measurement parameters, is essential to maximize the information obtained per unit time and the number of experiments performed. When optimization in advance is not possible because of limited prior knowledge of the system, autonomous, adaptive optimization must be implemented during the experiment. One approach to this involves sequential Bayesian optimal experimental design, which adopts mutual information as the utility function to be maximized. In this study, we applied this optimization method to the chemical exchange saturation transfer (CEST) experiment in nuclear magnetic resonance (NMR) spectroscopy, which is used to study minor but functionally important invisible states of certain molecules, such as proteins. Adaptive optimization was utilized because prior knowledge of minor states is limited. To this end, we developed an adaptive optimization system of ^15^N-CEST experimental conditions for proteins using Markov chain Monte Carlo (MCMC) to calculate the posterior distribution and utility function. To ensure the completion of MCMC computations within a reasonable period with sufficient precision, we developed a second-order approximation of the CEST forward model. Both simulations and actual measurements using the FF domain of the HYPA/FBP11 protein with the A39G mutation demonstrated that the adaptive method outperformed the conventional one in terms of estimation precision of minor-state parameters based on equal numbers of measurements. Because the algorithm used for the evaluation of the utility function is independent of the type of experiment, the proposed method can be applied to various spectroscopic measurements in addition to NMR, if the forward model or its approximation can be calculated sufficiently quickly.

## Introduction

One of the primary purposes of scientific experiments is to estimate parameters of mathematical models (henceforth referred to as model parameters) that represent or approximate systems of interest. Experimental design is optimized to satisfy specific statistical criteria, e.g., small variance in the estimated model parameters [1]. Bayesian design usually aims to maximize the amount of information gathered regarding the model and/or model parameters per unit cost. Thus, it adopts the expected gain in Shannon information in terms of the model parameters as the utility function to be optimized [2,3], which is equivalent to the expected Kullback–Leibler (KL) divergence as well as the mutual information [4–6]. Sequential or iterative design, which involves optimization based on recursive pairs of experimental and design phases, is expected to outperform pre-determined experimental designs in the case of nonlinear models as it leverages information obtained from previous observations [6–8]. On this basis, combined sequential and Bayesian design has also been suggested—however, this was difficult to be realized at the time of its proposal because of computational intractability [6]. Owing to methodological and hardware developments in recent years, the scope of this approach has expanded from simplified hypothetical problems to complicated real-world problems in various research fields [9–16].

To date, only a limited number of applications of sequential design have been reported in nuclear magnetic resonance (NMR) spectroscopy. High-Resolution Iterative Frequency Identification (HIFI) NMR was developed to estimate signal positions on original 3D spectra based on 2D projections via iterative selection of optimal projection angles [17]. Based on this method, an Assignment-directed Data collection Algorithm utilizing a Probabilistic Toolkit in NMR (ADAPT-NMR) was established to select informative angles for the stochastic assignment of protein NMR signals using a Bayesian network and belief propagation approach [18]. This approach calculated the utility function analytically to determine the optimal solution for networks containing loop structures [18]. However, it depended on ingenious model-specific treatments, thereby sacrificing generalizability. For simpler models with only two model parameters, Song et al. reported sequential optimization of experimental parameters for longitudinal or transverse relaxation measurements via exhaustive evaluation of the entire model parameter space [19]. Recently, an iterative selection method for evolution times and phases has been proposed in the context of non-uniform sampling for line-shape fittings. It utilizes linear approximation of a non-linear model [20]. Thus, the next step to expand the scope of NMR applications requires the development of sequential design methodologies capable of addressing relatively complicated nonlinear models in their intact forms, even at the cost of slightly higher computation loads.

Chemical shift exchange saturation transfer (CEST) is an NMR experiment that reveals minor and, therefore, directly invisible conformations of molecules, including proteins, slowly exchanging with a major visible state (Vallurupalli et al. [21] and references therein). Conventional CEST involving numerous sampling points corresponding to evenly spaced frequency offsets of saturation pulses is significantly time consuming [22,23]. To mitigate this problem, optimization of the offset step [23], interpolation of the sampling points in the frequency domain using linear prediction in the time domain [24], and multifrequency or cosine-modulated saturation pulses [25–27] have been proposed. In general, ^15^N-CEST experiments exhibit high signal-to-noise ratios (SNRs), because the typical protein concentration in ^15^N-CEST experiments is approximately 1 mM [21], with a few exceptions in the case of small peptides or relatively large (~10%) minor populations [28,29]. Therefore existing design approaches in the case of CEST assume sufficient sensitivity, thereby excluding repetition of the same experimental condition, analogous to the so-called “sampling-limited regime” in the context of non-uniform sampling or reduced dimensionality [30]. However, in the “sensitivity-limited regime,” repetitive sampling of important experimental conditions is essential to improve sensitivity via accumulation, leading to more precise model-parameter estimation. In the context of the preceding discussion, sequential Bayesian optimal design is a promising method because it samples informative experimental conditions preferentially.

In this study, a sequential Bayesian design method applicable to ^15^N-CEST experiments on proteins is proposed, assuming low SNR. To achieve this, we employed Markov chain Monte Carlo (MCMC) and Riemann sum approximations to calculate the utility function. As MCMC-based Bayesian analysis involves significantly more forward model evaluation times than point estimation analysis using a gradient-descent algorithm, we introduced a second-order approximation of the $R_{1\rho }$ relaxation coefficient to accelerate the evaluation of the forward model. We compared the precision and accuracy of the proposed and conventional methods based on model parameters estimated via simulation as well as real observation of the FF domain of the HYPA/FBP11 protein with the A39G mutation.

## Results and discussion

### Theoretical background of the proposed method

A detailed theoretical background of adaptive CEST is provided in Supporting Information. In this section, we present a brief summary. The pseudocode for adaptive CEST is as follows:

set the next experimental condition $\backslash {(}^{\left(1\right)}x$ to the reference ($\begin{array}{c} \omega_{RF}=0\text{H}z \end{array}$, $\begin{array}{c} \omega_{1}=0\text{H}z \end{array}$, $\begin{array}{c} T_{EX}=0\text{s} \end{array}$)
for iteration n,
  perform CEST measurements with the condition$\backslash {(}^{\left(n\right)}x$
  process NMR data to obtain intensities$\backslash {(}^{\left(n\right)}Y={\left\{{\left(n\right)y}_{k}\right\}}_{k=1}^{K}$
  sample model parameter ${\left\{\Theta^{\left(s\right)}\right\}}_{s=1}^{S}$ from the posterior distribution $\begin{array}{c} p\left(\Theta |𝒟\right) \end{array}$ via MCMC, where $\begin{array}{c} 𝒟={\left\{\left(i\right)Y\right\}}_{i=1}^{n} \end{array}$
  calculate the utility function U(x) using the MCMC samples
  set the next experimental condition $\backslash {(}^{\left(n+1\right)}x$, which maximizes U(x)
repeat iteration N times
if necessary, resample from the posterior distribution p(Θ|𝒟) for detailed analysis

For more details of the algorithm and the theoretical background, see S1 Text. The actual code used in this study is available on Open Science Framework at https://osf.io/3xwrp/ and RIKEN Research Data Management System (R2DMS) at https://dmsgrdm.riken.jp/9w25r/ as described in the Data Availability Statement.

The experimental condition x comprised the offset, $\omega_{RF}$, the strength, $\omega_{1}$, and the duration, $T_{EX}$. of the irradiation pulse. In general, a constant $T_{EX}$ is adopted in conventional CEST experiments [22]. However, based on the similarity between CEST and $R_{1\rho }$ experiments [31], the adaptive adjustment of $T_{EX}$ were considered in this study to improve the performance.

The model parameter, Θ, is a set of parameters corresponding to different residues, $\theta_{k}=\left\{p_{B}^{\left(k\right)},k_{ex}^{\left(k\right)},\omega_{B}^{\left(k\right)},R_{1}^{\left(k\right)},R_{2A}^{\left(k\right)},R_{2B}^{\left(k\right)},I_{0}^{\left(k\right)}\right\}$, where $p_{B}$ denotes the population ratio of the invisible (B) state, $k_{ex}=k_{on}+k_{off}$ denotes the exchange rate, $\omega_{B}$ denotes the chemical shift of the B state, $R_{1}$ denotes the longitudinal relaxation rate constant, $R_{2A}$ denotes the transverse relaxation rate constant for the observable major (A) state, $R_{2B}$ denotes that of the B state, and $I_{0}$ denotes the basal intensity at $T_{EX}=0$. We followed the assumption of Vallurupalli et al. [22] that the longitudinal relaxation rate of both states have the same $R_{1}$ value considering their finding that the CEST data does not contain information about that of the B state, $R_{1B}$. The chemical shift of the A state, $\omega_{A}$, is known from the spectrum. $p_{B}$ and $k_{ex}$ are likely to be constant or correlated among residues in single-domain globular proteins. However, this is usually confirmed by performing local (individual) fitting before global fitting, which fixes $p_{B}$ and $k_{ex}$ for all residues. In adaptive CEST, $p_{B}$ and $k_{ex}$ are also conservatively assumed to be independent of residues at the experimental design stage, whereas, in the detailed analysis after the experiment, the global model may be used.

As the utility function, we selected mutual information, defined by $U\left(x\right)=I\left(p\left(\hat{Y}\right);p\left(\Theta \right)\right)$, which is commonly used in Bayesian design [5,6]. Here, $\hat{Y}$ denotes the stochastic variable of the future observation, $\backslash {(}^{\left(n+1\right)}Y$. Mutual information is the expected value of the Kullback-Leibler (KL) divergence, $D_{KL}\left(p\left(\Theta |\hat{Y}\right)||p\left(\Theta \right)\right)$, over $p\left(\hat{Y}\right)$ [4–6]. As we assumed independence of $p\left(\theta_{k}\right)$ among residues, mutual information, $I\left(p\left(\hat{Y}\right);p\left(\Theta \right)\right)$, was calculated as the sum of residues, $\sum_{k=1}^{K}I\left(p\left({\hat{y}}_{k}\right);p\left(\theta_{k}\right)\right)$. The dimensions (i.e., the number of parameters) of Θ and $\theta_{k}$ are 7K and 7, respectively, as the evaluation of $\sum_{k=1}^{K}I\left(p\left({\hat{y}}_{k}\right);p\left(\theta_{k}\right)\right)$ requires K times of MCMC exploration of the seven-dimensional parameter space. However, this evaluation requires less computations than the evaluation of $I\left(p\left(\hat{Y}\right);p\left(\Theta \right)\right)$, which requires a single MCMC exploration of the 7K-dimensional parameter space. Note that the same x can be repetitively selected corresponding to different iterations, which may be effective in low-SNR cases, analogous to the increase in the number of scans in conventional NMR.

### Approximation of the CEST forward model with low computational cost

While performing autonomous measurements with a sequential Bayesian design, the posterior distribution and the utility function between the iterative measurements need to be evaluated within a short period to maximize the effectiveness of the NMR machine per unit time. Owing to the intrinsic similarities between CEST and $R_{1\rho }$ experiments, the CEST forward function has been approximated using $R_{1\rho }$ values in multiple papers, with reduced computational cost compared to that incurred in calculating the Bloch-McConnell equation completely [31,32]. While $R_{1\rho }$ can be calculated as one of the eigenvalues of the propagation matrix of the Bloch-McConnell equation, first-order approximations with some perturbations have been proposed to further reduce computational time [33,34]. However, as these approximations were not sufficiently accurate for our purpose, we approximated $R_{1\rho }$ to the second order (S2 Text, S4 Fig). When combined with the Palmer’s CEST approximation [31], the proposed $R_{1\rho }$ approximation method exhibited a typical computation time that exceeded those of first-order methods by only 25%, and was lower than that required for numerical eigenvalue calculation by an approximate factor of 30 (S3 Table).

### A simple 1-residue simulation

**Fig 1:**
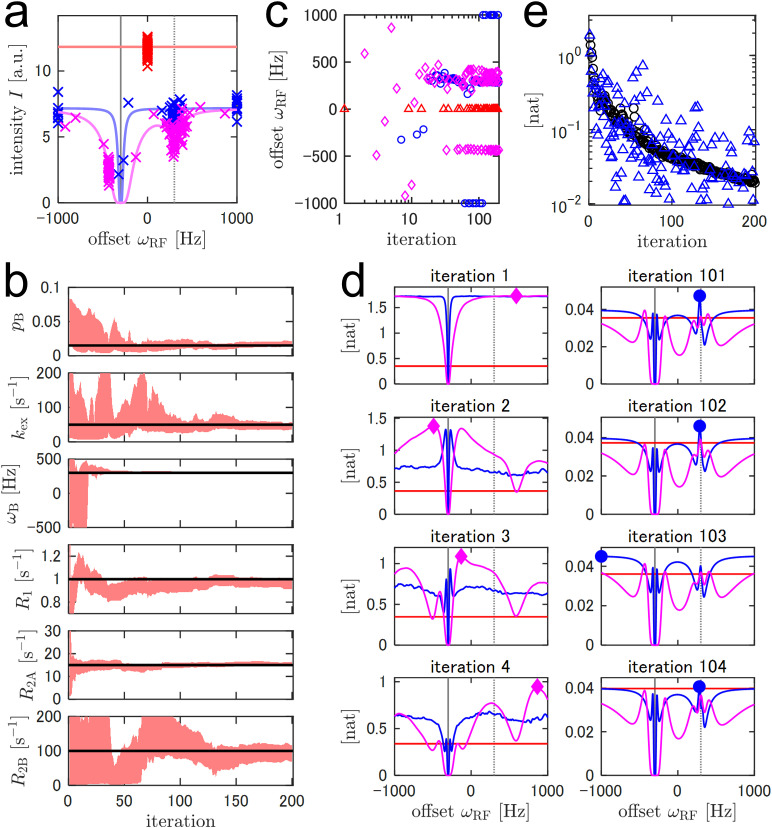
Simulated adaptive ^15^N-CEST with a single-residue virtual protein. (a) Observed signal intensities in all 200 iterations are indicated by crosses. The theoretical noiseless responses are indicated using lines. The colors represent different values of the irradiation strength, $\omega_{1}$: 0 Hz = red, 10 Hz = blue, and 50 Hz = magenta. Vertical solid and dotted gray lines represent the chemical shifts of A ($\omega_{A}$) and B ($\omega_{B}$) states, respectively. (b) Red areas represent 68.3% credible intervals (CIs) for the estimated model parameters. Horizontal black lines represent the actual parameter values. (c) The selected irradiation offsets, $\omega_{RF}$, are plotted indicating different values of $\omega_{1}$ using different markers: 0 Hz = red triangle, 10 Hz = blue circle, and 50 Hz = magenta diamond. (d) The mutual information evaluated following observations recorded at the designated representative iterations. The experimental condition with the highest mutual information is indicated—it is selected for the next iteration. The same markers as those used in (c) were used. $\omega_{A}$ and $\omega_{B}$ are indicated by vertical lines, as in (a). (e) The mutual information (black circle) and KL divergence calculated based on the realized observation (blue triangle).

As a proof-of-concept for adaptive CEST, we first simulated a 200-iteration experiment, henceforth referred to as simulation A1, using a virtual 1-residue protein (Fig 1a). The experimental configurations and true model parameters are listed in Supporting Information (S6 Table and S7 Table). The SNR, defined to be $\frac{I_{0}}{\sigma }$, was taken to be 20, where σ denotes the standard deviation of the spectral background noise. The apparent SNR of the CEST curve was reduced to 12.2 when $\begin{array}{c} T_{EX}=0.5\text{s} \end{array}$ because the off-resonance signal intensity was $I_{0}\exp\left(-R_{1}T_{EX}\right)$. There were 803 experimental condition candidates (𝒳), including the reference ($\begin{array}{c} T_{EX}=0\text{s} \end{array}$) for the simulation A1. The offset $\omega_{RF}$ spanned from -1000–1000 Hz, and the strength $\omega_{1}$ was either 10 or 50 Hz.

Fig 1b illustrates the 68% CIs of the model parameters plotted with respect to varying numbers of iterations. Unsurprisingly, the estimation of model parameters became more precise as the number of iterations was increased. During the first 20 iterations, there was almost no information regarding the chemical shift of the B state. After that, it was confirmed that $\omega_{B}$ was ~300 Hz, and prompting us to gather information about other exchange parameters ($p_{B}$, $k_{ex}$, $R_{1}$, $R_{2A}$, and $R_{2B}$). This change also appeared during the selection of the experimental conditions. In the former iterations, the selected offset values were varied to search for a B-state dip, whereas in the later iterations, the offset values were more stable (Fig 1c). The selection of the experimental conditions during the later iterations was qualitatively explained using the simulated CEST curves. Slight changes in $p_{B}$ and $k_{ex}$ values affected the dip size of the B state (S8 Fig). This indicated that the B-state on-resonance experiments ($\begin{array}{c} \omega_{RF}≅\omega_{B}=300\text{H}z \end{array}$) were selected because they were informative for $p_{B}$ and $k_{ex}$. Similarly, the slight off-resonance corresponding to the B-state ($\begin{array}{c} \omega_{RF}≅400\text{H}z \end{array}$) experiments was for $R_{2B}$; the slight off-resonance corresponding to the A-state ($\begin{array}{c} \omega_{RF}≅-450\text{H}z \end{array}$) experiments was for $R_{2A}$; the off-resonances corresponding to $\omega_{RF}=-1000$ or $\begin{array}{c} 1000\text{H}z \end{array}$ was for $R_{1}$, and the reference experiments were for $I_{0}$. However, because some of the model parameters were correlated, information on one parameter aided the estimation of others (S9 Fig).

**Fig 2:**
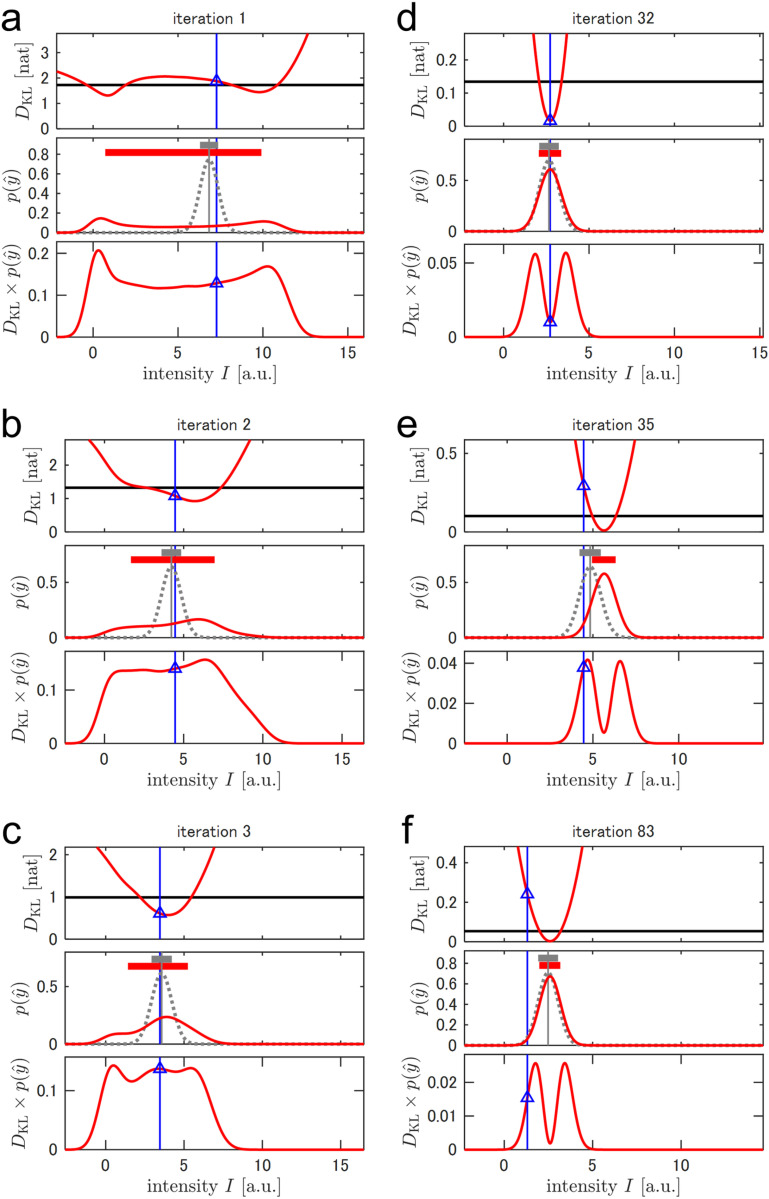
A breakdown of mutual information as the expected KL divergence and its difference with the realized KL divergence in single-signal simulation. In each panel, the mutual information at the designated iteration and the KL divergence calculated after the observation at the following iteration are depicted. The red line at the top represents the KL divergence, $D_{KL}\left(p\left(\theta |\hat{y}\right)||p(\theta )\right)$, plotted with respect to the future observation, $\hat{y}$. The red line in the middle indicates the estimated $p\left(\hat{y}\right)$ calculated based on the current knowledge plotted against $\hat{y}$. The gray vertical line indicates the ideal noiseless response calculated using the true parameters. The gray dotted line represents the true distribution with a random observation noise. The red and gray horizontal thick lines represent the 68.3% CI of the estimated and the true $p\left(\hat{y}\right)$, respectively. The product of the above two functions of $\hat{y}$, $D_{KL}\left(p\left(\theta |\hat{y}\right)||p(\theta )\right)\times p\left(\hat{y}\right)$, is represented by the red line at the bottom. As KL divergence estimates the degree of knowledge update after each subsequent observation, it tends to increase significantly when the subsequent observation is unexpected. Over the first iterations (i.e., at Iteration 1, 2, and 3), the knowledge about the model parameters is limited; thus, the estimated range of $p\left(\hat{y}\right)$ is wide. In contrast, at later iterations (e.g., at Iteration 32, 35, and 83), the range of the estimated $p\left(\hat{y}\right)$ reaches a width equal to the observation noise. As a result, the product, $D_{KL}\left(p\left(\theta |\hat{y}\right)||p(\theta )\right)\times p\left(\hat{y}\right)$, exhibits trapezoidal or bimodal shapes in the former and latter cases, respectively. Mutual information, represented here by a black horizontal line, is the integration of the product, $\int D_{KL}\left(p\left(\theta |\hat{y}\right)||p(\theta )\right)p\left(\hat{y}\right)d\hat{y}$. Blue vertical lines in all plots indicate the realized observation at each successive iteration. The blue triangles in the top and the bottom panels represent the corresponding realized $D_{KL}\left(p\left(\theta |\hat{y}\right)||p(\theta )\right)$ and $D_{KL}\left(p\left(\theta |\hat{y}\right)||p(\theta )\right)\times p\left(\hat{y}\right)$, respectively.

Fig 1d depicts the mutual information as the utility function for representative iterations. For the first four iterations, the shape of the function varied drastically, suggesting that the posterior distributions, especially of $\omega_{B}$, were considerably updated by informative observations (Fig 1d: left panel). Consequently, various offset values were selected to search for the B state dip during these iterations (Fig 1c). In contrast, after reaching confidence for $\omega_{B}$, the shape of the function was stable with $\omega_{RF}$ being restricted to the aforementioned typical conditions (~300 Hz, ~400 Hz, ~−450 Hz, off resonances, or the reference) at these iterations (Fig 1d right). As discussed in the Theory section, mutual information is expected to exhibit KL divergence over the observation distribution, $p\left(\hat{Y}\right)$. Fig 1d depicts the mutual information evaluated prior to observation and the KL divergence evaluated using the realized $\hat{Y}$ after observation. The mutual information decreased as the number of iterations increased (Fig 1e). This was attributed to a general lack of new information obtained from a single future observation compared to the knowledge pieced together based on numerous past observations as the number of iterations increased. In the first few iterations, the probability density of $p\left(\hat{Y}\right)$ was widespread, indicating the difficulty of prediction (Fig 2a-c). In such cases, the system confirmed unpredictable observations, as explained in the literature, where the observations were considered to be virtually free from noise [35]. However, this explanation no longer held true after the prediction of uncertainty was reduced to the noise level. In this case, the smaller KL divergence compared to that of mutual information reduced the difference between the posterior distributions before and after the observation. For example, during Iteration 32, 68% CI of the intensity of the subsequent observation was predicted to be 2.06–3.37 (Fig 2d). Because the observation at the Iteration 33 fell within the predicted range (2.73), the KL divergence was as small as 0.0167 nat, which was lower than the mutual information of 0.134 nat (Fig 2d). Such well-predicted measurements increased confidence in current knowledge. In contrast, KL divergences larger than the mutual information indicated an outlying observation derived from significant noise and/or inaccurate prediction. The former case was instantiated in Iteration 83, which featured large KL divergence owing to accidental large noise, even though the prediction of the observation was close to the true distribution (Fig 2f). The latter case was instantiated in Iteration 35. The 68% CI of the subsequent observation was 4.95–6.32, whereas the true value was 4.46. The disparity between the predicted range and the observation led to a larger KL divergence of 0.293 nat compared to the mutual information of 0.101 nat, as the observed divergence was significantly smaller than the predicted divergence (Fig 2e). Such informative observations increase the agreement between the knowledge gathered and the true values of the model parameters. As mutual information is defined as the expected value of KL divergence, $I\left(p\left(\hat{Y}\right);p\left(\Theta \right)\right)={𝔼}_{\hat{Y}}\left[D_{KL}\left(p\left(\Theta |\hat{Y}\right)||p\left(\Theta \right)\right)\right]=\int p\left(\hat{Y}\right)D_{KL}\left(p\left(\Theta |\hat{Y}\right)||p\left(\Theta \right)\right)d\hat{Y}$, the possibility of large knowledge multiplied with its probability updates contributes the mutual information. In other words, the system autonomously designs experiments to decrease the risk of misevaluating the model parameters.

### Simulation with different irradiation durations

In conventional CEST, irradiation pulses are usually applied with various offsets, $\omega_{RF}$, a single or several strengths, $\omega_{1}$, and a fixed duration, $T_{EX}$ [22]. This simple experimental configuration is beneficial to both experimental design as well as analysis of the results. However, we varied $T_{EX}$ to improve parameter estimations, motivated by the close resemblance between CEST and $R_{1\rho }$ experiments, where $T_{EX}$ is varied while investigating $R_{1\rho }$ via curve fitting [31]. Because the design and analysis steps of the adaptive experiment are free from human instruction, increasing the dimensionality of the design space from two to three is acceptable.

**Fig 3:**
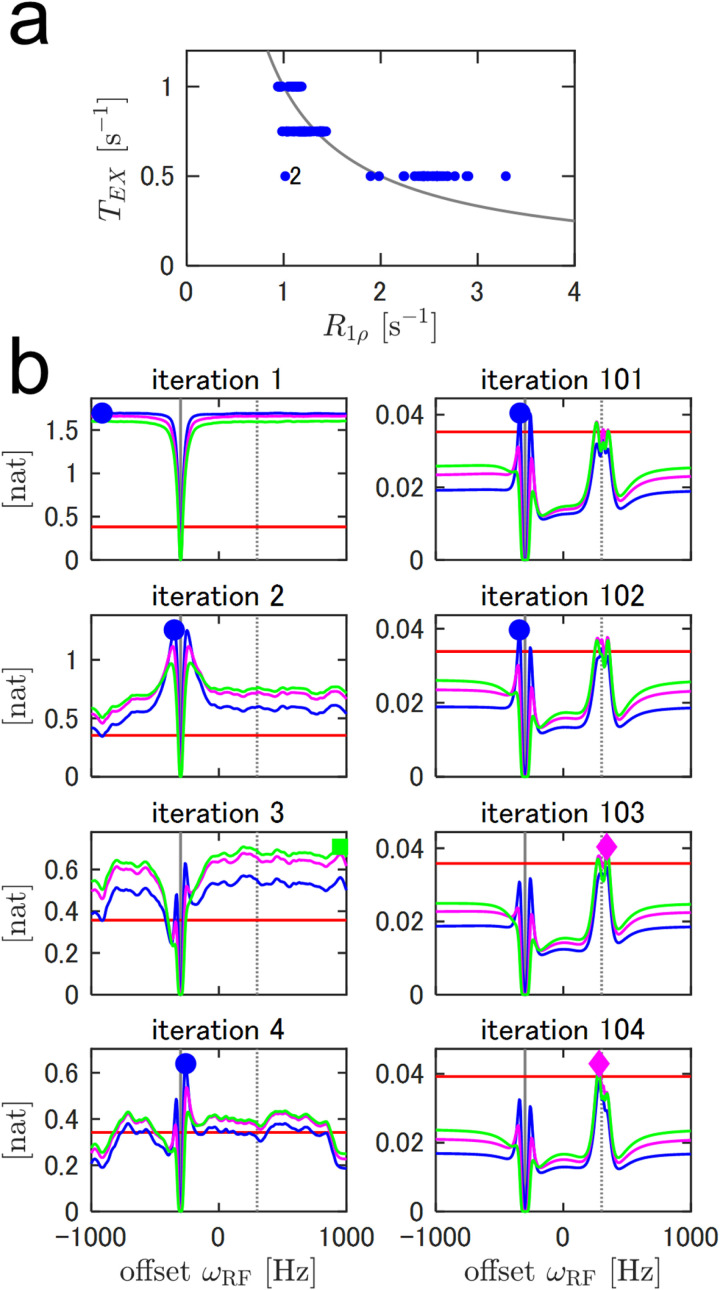
Adaptive CEST simulation of the virtual single-residue protein with variable irradiation durations, $T_{EX}$. (a) Selected $T_{EX}$ values with respect to maximum-a-posteriori $R_{1\rho }$. The gray line represents $T_{EX}=\frac{1}{R_{1\rho }}$. An outlier is observed at iteration 2, indicated by the letter “2”. (b) Mutual information at several representative iterations. The red, blue, magenta, and green lines correspond to $\begin{array}{c} T_{EX}=0\text{s} \end{array}$ (reference), $\begin{array}{c} 0.50\text{s} \end{array}$, $\begin{array}{c} 0.75\text{s} \end{array}$, and $\begin{array}{c} 1.00\text{s} \end{array}$, respectively. The different markers represent selected experimental conditions with the highest mutual information.

To exemplify incorporating $T_{EX}$ as an experimental parameter, we tested a single-residue simulation named A2 with the same configuration as the simulation A1, except for fixed $\omega_{1}$ and variable values of $T_{EX}$ over 0.50, 0.75, and 1.00 s (S6 Table). The model-parameter estimation was less precise than that for A1 (S10 Fig), possibly due to the lack of variance of $\omega_{1}$ [21,24]. For precise estimation of relaxation constants such as $R_{1\rho }$, $T_{EX}=\frac{1}{R_{1\rho }}$ was observed to be the most informative sampling point when $I_{0}$ was known. The selected value of $T_{EX}$ was close to the inverse of the estimated $\frac{1}{R_{1\rho }}$, with some exceptions possibly derived from the uncertainty of $R_{1\rho }$ and $I_{0}$ estimations (Fig 3a). When the $R_{1\rho }$ estimate was large, smaller values of $T_{EX}$ increased reliability. However, the approximation of the CEST forward model used in this study assumed sufficiently long $T_{EX}$ to eliminate transverse magnetization in the tilted reference frame [31]. This was also true in case of the Bloch-McConnell equation because the complete measurement of $B_{1}$ inhomogeneity is difficult [36]. For these reasons, we set the lower limit of $T_{EX}$ as 0.5 s to ensure the accuracy of forward calculation. The curves of the utility function corresponding to different $T_{EX}$ were similar even in the first iterations (Fig 3b), in contrast to those corresponding to different $\omega_{1}$ in the simulation A1 (Fig 1e). Therefore, we decided to use only $T_{EX}$ = 0.5 or 1.0 s as candidates for the following simulations and measurements, conserving computational resources for the more important $\omega_{1}$ variation.

### Simulation involving a virtual 70-signal protein

**Fig 4:**
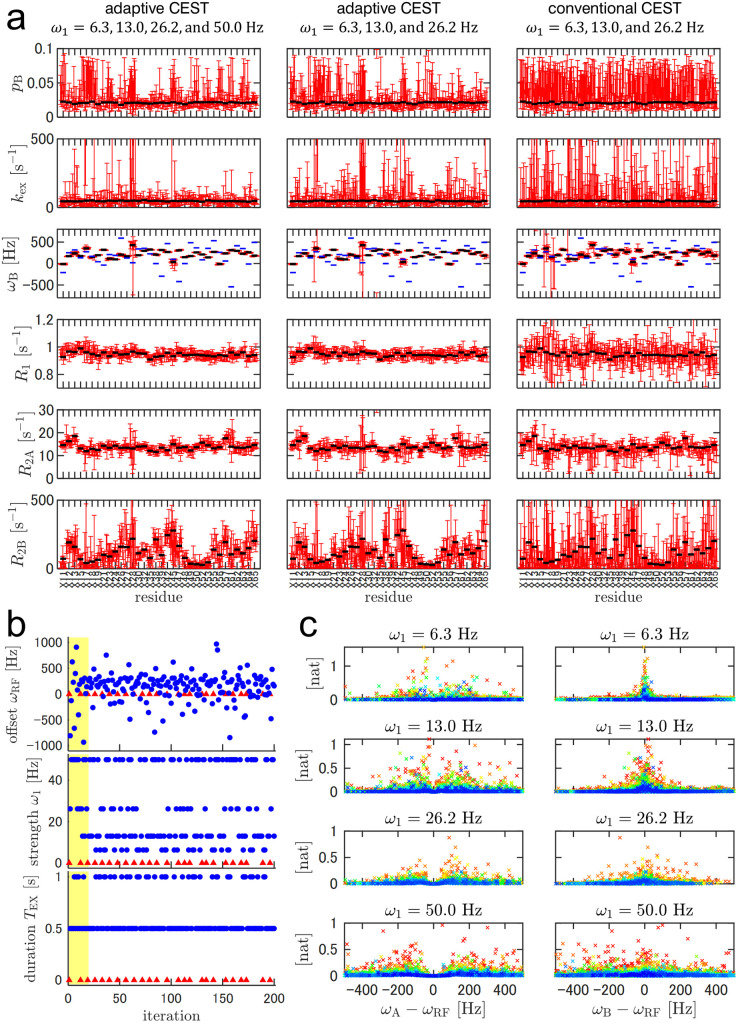
^15^N-CEST simulations with a virtual 70-signal protein. (a) Model parameter estimation after 192 iterations or 192 2D measurements. The left, middle, and right panels correspond to adaptive CEST with $\omega_{1}=6.3$, 13.0, 26.2, and 50.0 Hz; adaptive CEST with $\omega_{1}=6.3$, 13.0, and 26.2 Hz; and a conventional CEST with $\omega_{1}=6.3$, 13.0, and 26.2 Hz; respectively. The red lines represent the 68.3% CIs of the model parameter estimates. For each residue, 10 individual simulations with different random seeds are performed and plotted. The horizontal black lines represent the true values of the parameters. The horizontal blue lines in the $\omega_{B}$ plots represent $\omega_{A}$. Terminal residues and residues with $\begin{array}{c} \left|\omega_{B}-\omega_{A}\right|<100\text{H}z \end{array}$ are omitted. Plots with all residues are included in Supporting Information. (b) The selected experimental condition for adaptive CEST simulation with $\omega_{1}=6.3$, 13.0, 26.2, and 50.0 Hz. Blue circles and red triangles correspond to non-reference and reference experiments, respectively. The first 20 iterations are highlighted in yellow. (c) Mutual information of adaptive CEST simulation with $\omega_{1}=6.3$, 13.0, 26.2, and 50.0 Hz plotted against $\omega_{A}-\omega_{RF}$ and $\omega_{B}-\omega_{RF}$. Only Iterations 21–192 are illustrated, colored by a spectrum ranging from red (at Iteration 21) to blue (at Iteration 192).

In practical ^15^N-CEST experiments on proteins, we observed multiple signals corresponding to all residues, except prolines and an N-terminal residue. We defined the utility function to be the mutual information in the 7K-dimensional model parameter space, i.e., the sum of the mutual information of individual K signals (see S1 Text for details). In this situation, because the same experimental conditions were applied to signals with potentially different model parameters at different iterations, the conditions may not have been optimal for each signal. To evaluate the performance of multiple signals, we simulated adaptive (A3 and A4) and conventional (C4) CEST using a virtual 70-signal protein (S6 Table). The number of 2D measurements was set to 192 for all simulations to ensure a fair comparison of their performances based on equal instrumental resources. For adaptive CEST,$\omega_{1}$ candidates were either 6.3, 13.0, 26.2, and 50 Hz (for A3) or 6.3, 13.0, and 26.2 Hz (for A4). For conventional CEST, $\omega_{RF}$ values comprised 63 evenly spaced points between -1000 and 1000 Hz (32.3-Hz step); $\omega_{1}$ values were the same as in A4; and $T_{EX}$ was fixed to 0.5 s. Including the three references ($\begin{array}{c} \omega_{1}=0\text{H}z,T_{EX}=0\text{s} \end{array}$), 63×3+3=192 2D measurements were performed in aggregate. We also performed conventional CEST simulations C5–C9 with double or single $\omega_{1}$ values (S6 Table). All simulations were repeated ten times with different random seeds. Both conventional and adaptive CEST data were subjected to Bayesian analysis to compare the uncertainties of model parameters. Both the precision and accuracy of model parameter estimation for adaptive CEST were better than or comparable to those for conventional CEST corresponding to most signals (Fig 4a, S12 Fig). Notably, the lower bound of the CIs of $R_{2B}$ was higher in adaptive CEST. i.e., more information was obtained about $R_{2B}$. One of the key features of adaptive CEST is that it does not require prior knowledge of the $\omega_{B}$ distribution, which is generally unknown in CEST experiments. Based on a set of candidate experimental conditions with broad offsets, adaptive CEST automatically focuses on the most informative ones based on the acquired data. In contrast, conventional CEST requires a predetermined offset range, often requiring broader selection than necessary. To evaluate hypothetical scenarios where prior knowledge is available, e.g., that most $\omega_{B}$ values fall within the [0, 500] Hz range, we conducted additional simulations using conventional CEST with $\omega_{RF}$ limited to [0, 500] Hz, as illustrated in C10 and C11 of S12 Fig. As expected, parameter estimation was less presice corresponding to residues with $\omega_{A}$ or $\omega_{B}$ values lying outside the aforementioned range. However, for other residues, adaptive CEST continued to provide more precise or comparable parameter estimates compared to conventional CEST.

Owing to variations in $\omega_{A}$, $\omega_{B}$, and the other model parameters among the signals, the optimum experimental condition depended on the signal. However, adaptive CEST optimized the experimental design notwithstanding the variety of optima. As in the case of the 1-signal simulation A1, the selected experimental condition for A3 over the first ~20 iterations was varied to investigate B-state chemical shifts (Fig 4b). During this stage, strong irradiation ($\begin{array}{c} \omega_{1}=50\text{H}z \end{array}$) was preferred for effective exploration with a wide irradiation range. Over the rest of the iterations, both strong ($\begin{array}{c} \omega_{1}=50\text{H}z \end{array}$) and weak ($\omega_{1}=6.3$ or $\begin{array}{c} 13.0\text{H}z \end{array}$) strengths were selected. The mutual information of the individual residues plotted against $\omega_{A}-\omega_{RF}$ or $\omega_{B}-\omega_{RF}$ indicated that stronger irradiation facilitated the collection of information from a wide range of signals while weaker irradiation facilitated the collection of more detailed information corresponding to fewer signals (Fig 4c). Adaptive CEST was autonomously balanced using these two types of irradiation by following the definition of the utility function. It should be noted that the use of both strong and weak irradiation is important for adaptive CEST for this reason, together with the reported importance of exchange parameter estimation in conventional CEST [21,24]. To investigate whether more iterations are required to reach certain model-parameter precision when multiple residues are targeted, the precision of representative residues were plotted against the iteration number (S13 Fig). Although this simulation should not be directly compared to the one-signal simulation A1 (Fig 1b) due to differences in the model parameters and experimental conditions, the precision of the model parameters apprears to largely differ between signals, some of which reached comparable precision to the one-signal case at early iterations. This difference likely results from the variance in the SNRs, chemical shifts, and/or exchange parameters. In contrast to the remaining residues, A3 and A4 underperformed corresponding to residue X28 (Fig 4a), possibly owing to its outlying $\omega_{B}$ value. The proposed adaptive CEST autonomously prioritized estimation for the majority of signals, rather than for a single outlying signal based on the definition of the utility function. To focus on a specific signal, a different definition of the utility function should be used, e.g., residue-specifically weighted mutual information. Moreover, additional iterations with different utility functions would be useful for gathering more information about a specific residue after the experiment. Simulation A3, followed by an additional 48 iterations using the new utility function, i.e., the mutual information of residue X28, outperformed the simulation with the same number of iterations using the same utility function in terms of parameter estimation for residue X28 (S14 Fig).

### Evaluation using real measurements

**Fig 5:**
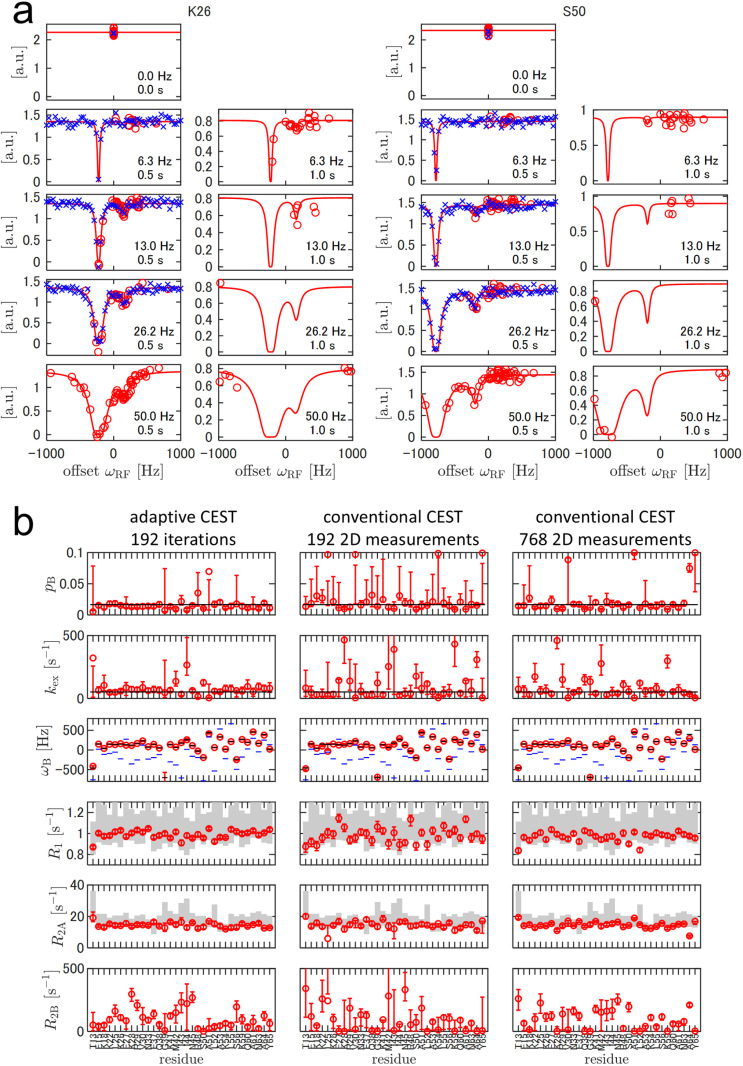
Adaptive ^15^N-CEST experiment with a 0.1 mM FF A39G protein, compared with conventional experiments. (a) Observed intensities of the two representative residues in the adaptive experiment (red circle) and the conventional experiment (blue cross). $\omega_{1}$ and $T_{EX}$ values are indicated in each plot. Red curves represent the responses calculated using the MAP estimator after 192 iterations of the adaptive experiment. (b) Estimated model parameters of adaptive CEST with 192 iterations (left), conventional CEST with 192 2D measurements (middle), and conventional CEST with 768 2D measurements (right). The red circles and error bars represent the MAP estimator and 68.3% CIs, respectively. The black horizontal lines represent the reported parameters from conventional CEST with 2.0 mM protein [22]. The gray bars indicate 68.3% CIs from the separately recorded ^15^N relaxation experiments with the same 0.1 mM sample.

**Fig 6:**
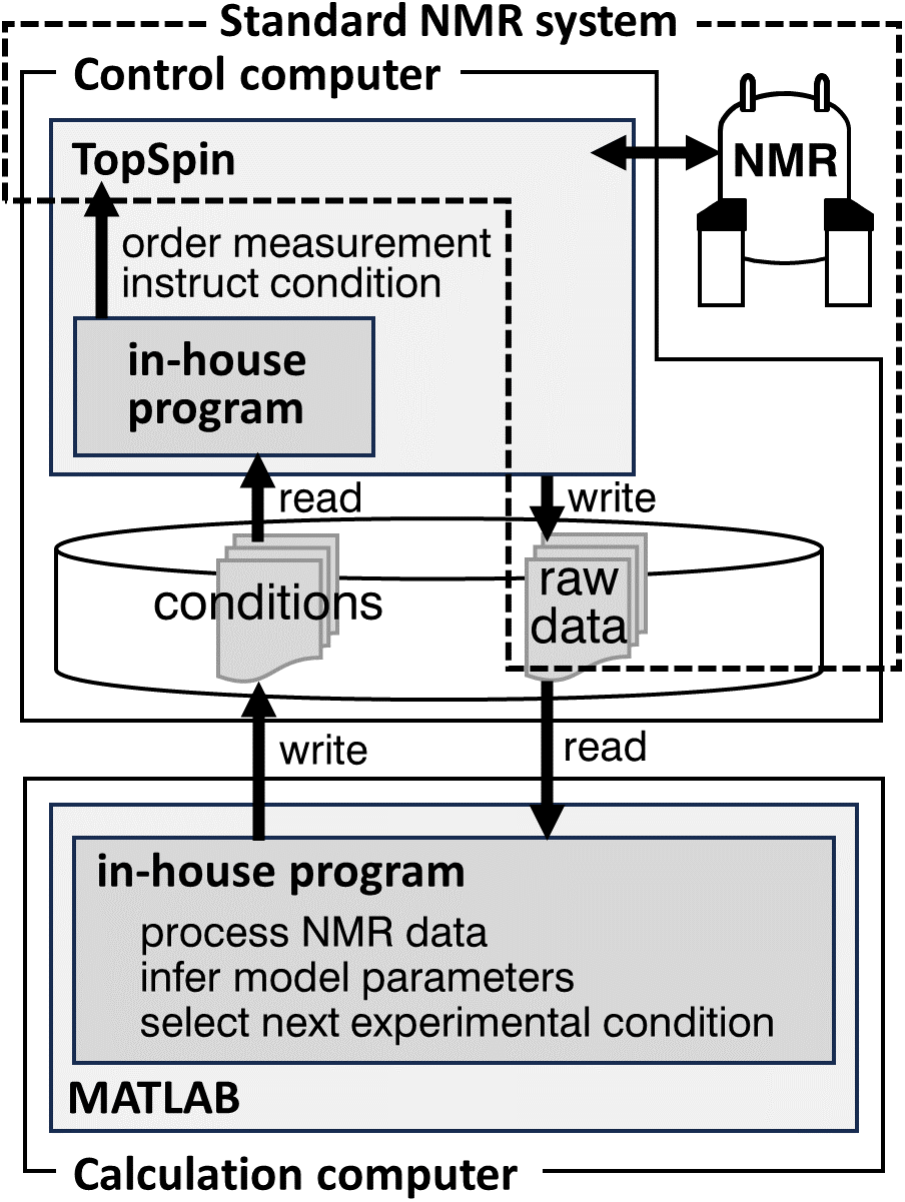
Schematic representation of the experimental configuration for adaptive CEST.

Finally, we performed a real CEST experiment with the 71-aa FF domain of the HYPA/FBP11 protein with A39G mutation [22] (Fig 5a) using the same experimental configurations as those used in simulations A3 and C4. For adaptive CEST, MCMC and mutual information calculations were performed on a remote computer, which transmitted messages of selected experimental conditions to an NMR-control computer to run iterations (Fig 6, see experimental section for details). We employed this configuration because the NMR-control computer provided by the NMR vendor had insufficient computational performance to calculate the experimental conditions involving many MCMC iterations. If the NMR-control computer has adequate computational capabilities, the program can be executed on this computer. The measurement time for a single experimental condition, i.e., a single 2D spectrum, was 19 and 24 min for $T_{EX}=0.5$ and $\begin{array}{c} 1.0\text{s} \end{array}$, respectively. The typical computation times for MCMC and mutual information were 50–60 s and 18 s, respectively, using dual Intel Xeon E5-2690v4 CPUs (a total of 28 physical cores). In combination with the communication time, the typical turnaround time between NMR measurements was less than 90 s, which slightly increased the total occupation time of the machine.

Fig 5b illustrates the model parameter estimates after 192 iterations of 2D measurements. The accuracy was evaluated with respect to the values of $p_{B}$, $k_{ex}$, and $\omega_{B}$ reported in the literature, separate inversion recovery or CPMG experiments for $R_{1}$, $R_{2A}$, and conventional CEST with 4-fold more scans for all parameters (Fig 5b). Adaptive CEST estimates agreed with these values. It outperformed, or at least performed comparable to, conventional CEST in terms of precision, except in the case of G39, which exhibited an outlying $\omega_{B}$ value.

## General discussion

In the preceding discussion, we assumed that the forward model and the noise were known. However, the proposed Bayesian experimental design method can also be implemented in general NMR experiments or even other applications by simply substituting the forward and noise models, while retaining calculation of mutual information using MCMC. As described in the Introduction, experimental designs pursuing the best inference of model parameters have been proposed in various research fields [9–16]. The method proposed in this paper serves as another example of a Bayesian optimal sequential experimental design, in which the computation algorithm does not depend on the type of measurement. Its applicability to NMR CEST measurements was validated by introducing an approximation for the forward model compatible with relatively heavy MCMC computations. One possible drawback of this method is its dependence on forward models. The proposed system considers a two-state forward model; thus, the system does not attempt to search for a third state after finding the second one. This is problematic in certain ^15^N-CEST applications involving models with more than two states; e.g., the observation of intermediate states in fold-unfold equilibria [37,38]. In principle, adaptive CEST could be extended to accommodate three-state exchange explicitly, but this would require developing a new approximation method tailored for the three-state exchange model. It should be noted that, in addition to general human-instructed research, it is possible to switch the model used for analysis after data acquisition. In this case, following adaptive measurement, all data are analyzed using a three-state model, expecting some information on the third state, despite the autonomous system not actively gathering such information. If the SNR is low, as assumed in this study, it is practical to prioritize identifying only one minor state over identifying all.

In most practical applications of conventional CEST, manual recognition of minor state dips is easy when measurements are equally spaced and the SNR is high. However, under low SNR conditions, distinguishing minor dips from noise becomes challenging. One key advantage of Bayesian analysis is its objectibity—unlike manual interpretation, it outputs probability distributions based on computational analysis, enabling more reliable identification of minor states.

The optimal number of iterations depends on the trade-off between available machine time and the required precision, complicating the establishment of a universal guideline. However, as demonstrated in our example (Fig 5b), adaptive CEST performed comparably to conventional CEST based on only a quarter of the number of 2D measurements. This efficiency stems from its ability to allocate measurement resources to the most informative experimental conditions. As a rough reference, this four-fold reduction can be used to estimate the number of required iterations, even though the actual number may vary depending on factors such as protein size, the distribution of $\omega_{A}$ and $\omega_{B}$, and/or SNR. Notably, when the protein concentration is low adaptive CEST achieved acceptable precision values, but required more iterations than practical CEST experiments under high-SNR conditions.

Theoretically, adaptive CEST could be impremented using a global model by assuming $p_{B}$ and $k_{ex}$ to be identical for all residues and optimizing the experimental design on this basis. In this scenario, precise estimation of $p_{B}$ and $k_{ex}$ based on a subset of residues could enable adaptive CEST to deprioritize measurements in determining $p_{B}$ and $k_{ex}$ for other residues. However, as mentioned in the main text, implementing adaptive CEST under a global model presents significant challenges due to the infeasibility of calculating the posterior distribution and mutual information within the short time intervals between successive NMR measurements. Even if feasible, this approach risks missing data that could reveal residue-specific variations in $p_{B}$ and $k_{ex}$, which is generally undesirable in most protein CEST applications. Therefore, in this study, we designed experimental conditions assuming variation in $p_{B}$ and $k_{ex}$ across residues and collected the data accordingly. Subsequently, we performed a comprehensive analysis of the complete dataset. If the results suggest that $p_{B}$ and $k_{ex}$ are identical for all residues or over a subset of residues, the dataset can be re-analyzed under these global constraints. However, this re-analysis currently relies on fitting-based methods rather than Bayesian-based ones, owing to the unavailability of the Bayesian CEST program that supports global models.

The utility function for adaptive CEST is defined to be the sum of the mutual information across all residues. Consequently, when multiple residues favor a particular condition, their preference is more likely to be selected, leading to more precise parameter estimation for residues with dominant $\omega_{A}$ and $\omega_{B}$ values, although at the cost of reduced precision for outliers. However, in proteins, residues within the same domain or in close proximity often exhibit cooperative motions. As a result, prioritizing the majority still allows for robust extraction of key parameters, such as $p_{B}$, $k_{ex}$, and cooperative dynamics. In general, glycine residues are more likely to be outliers due to their up-field ^15^N random coil shifts.

To acquire responses corresponding to various frequency offsets efficiently, multifrequency or cosine-modulated saturation pulses have been used in some studies, instead of conventional single-frequency pulses [25–27]. The proposed method is capable of integrating these conventional and advanced pulses by considering them as different experimental condition candidates, provided that the forward model computation for advanced pulses is compatible with the MCMC calculations. The performance of the integrated method is expected to be better than or at least comparable to that of adaptive CEST with a conventional pulse.

The parameter range to be explored should be defined to cover the possible parameter range of the system. If it is found that the range is too narrow after the experiment, the data can be re-analyzed using a wider parameter range, although the data acquisition may be based on nonoptimal experimental design. In this study, we retrospectively confirmed the appropriateness of the parameter range based on the posterior distributions (Figs 1b, 4a, 5b, S9, S10, and S14). Although a wider parameter range can more reliably cover the possible parameter space, a narrower range of parameters is preferable for two reasons. The first is to allow the MCMC system to find the global minimum within a limited number of MCMC iterations to reduce the NMR idle time. Narrowing the $R_{1}$ range is unnecessary since its broader inclusion does not impact MCMC convergence negatively, given the monotonic response of measured intensity with respect to $R_{1}$ variation (S8 Fig). The second is to ensure fast computation while maintaining the accuracy of the approximation. In general, an approximation that is applicable to a wider range of parameters requires a longer computation time. Among the model parameters of CEST, adequate coverage of the estimated $\omega_{B}$ range by the $\omega_{RF}$ range, as well as the offsets of experimental condition candidates and MCMC exploration ranges for $\omega_{A},\omega_{B}$, and $\omega_{RF}$ is essential. This should be determined based on prior knowledge of the $\omega_{B}$ distribution and the magnetic field strength. Although we used the range $\begin{array}{c} -1,000\text{H}z\leq \omega_{RF}\leq 1,000\text{H}z \end{array}$ in this study, our results indicate that the approximation remains sufficiently accurate even when extended to the range $\begin{array}{c} -5,000\text{H}z\leq \omega_{A},\omega_{B},\omega_{RF}\leq 5,000\text{H}z \end{array}$. Besides $\omega_{RF}$, we believe that the proposed approximation and the parameter range used in this study constitute a balanced configuration that may be applicable to various practical systems.

In this study, the 2D measurement time varied with $T_{EX}$. As we did not consider this variation over a single iteration, the objective of the experimental design was the amount of information gathered per iteration, not per unit time. If the measurement time is considered, the mutual information per unit measurement time may be a more suitable utility function. In this study, the NMR spectrometer was idle between measurements. It is worth considering to acquire data during this period as well, e.g., using 1D ^15^N-HSQC to monitor the state of the sample.

As discussed previously, in some situations, extending the iterations with different utility functions is useful. If, upon completion, an experiment yields insufficient general precision for model parameter estimates, more iterations can be added after the adaptive experiment using the same utility function. In this study, we performed the experiments with a fixed number of iterations; in this context, autonomous halting or extension of the experiment based on the required precision is an interesting topic.

Furthermore, we considered adaptive CEST with four different $\omega_{1}$ values and two different $T_{EX}$ values. We did not attempt to wield more precise control over these parameters because the computation time for mutual information in the current algorithm was proportional to the number of discrete experimental conditions. In future works, a suitable experimental configuration should be identified for adaptive CEST.

The choice between adaptive CEST and conventional CEST depends on the desired balance between measurement resources and precision. For example, if the minimum number of scans required for phase cycling is 8, which needs to be increased to 16 due to low SNR, adaptive CEST functions advantageously by maintaining 8 scans per experiment while doubling the number of 2D measurements (i.e., iterations). This approach enables the allocation of limited measurement resources to the most informative experimental conditions. However, if the $\omega_{A}$ and $\omega_{B}$ distributions are broad and spread uniformly over the offset range, the advantage of adaptive CEST over the conventional alternative diminishes. When applied to larger proteins, more signals are expected to occupy more diverse chemical-shift spaces. This may reduce the possibility of optimizing the experimental conditions, and this issue will be investigated a future study. Adaptive CEST may also reduce the number of measurements in a sampling-limited regime with high SNR, although this scenario has not been tested explicitly. In such high-SNR conditions, simpler experimental design algorithms that do not require Bayesian estimation might be more suitable.

## Conclusions

The primary advantage of the adaptive optimization methods proposed in this paper is that it can select effective experimental conditions for model parameter estimation without human intervention, provided that a reliable forward model is available. In CEST, one of the model parameters ($\omega_{B}$) is almost unknown before starting the experiment, even though the design of the other parameters depends on it. In such cases, autonomous experimental design is helpful because of the independence of external instruction that it provides while switching the target model parameters. Moreover, for complicated nonlinear model functions, manual estimation of the current knowledge based on in-hand data, accurate prediction of the behavior of the observation with respect to varying model parameters, and appropriate experimental design to maximize information gathered about model parameters are difficult.

## Materials and methods

### Sample preparation

The gene encoding the FF domain of HYPA/FBP11 containing the A39G mutation was synthesized by Eurofins Genomics K.K. (Tokyo, Japan). A template DNA encoding FF A39G with a histidine affinity tag and a Tobacco Etch Virus (TEV) protease cleavage site at its N-terminus was constructed using a polymerase chain reaction (PCR)-based method, as described previously [39]. U-^13^C/^15^N-labeled FF A39G was produced using the dialysis mode of a cell-free protein synthesis system, with a 9-mL inner reaction solution [40–43]. Affinity purification was performed by loading the reaction solution diluted with buffer A (20 mM Tris-Cl pH 8.0, 500 mM NaCl, and 20 mM imidazole) onto a HisTrap 1-mL affinity column (Global Life Sciences Solutions, Marlborough, MA, USA) and eluting with buffer B (identical to A, except containing 500 mM imidazole). The eluate was exchanged with buffer A via ultrafiltration with Amicon Ultra 15 MWCO = 3K (Merck, Darmstadt, Germany) and subsequently cleaved with 100 ug of TEV for 16 hours at 25 °C. The cleaved product was further purified using a HiTrap SP 1-mL cation exchange column (Global Life Sciences Solutions, Marlborough, MA, USA) with a gradient of C (50 mM 2-(N-morpholino)ethanesulfonic acid (MES)-sodium, pH 6.0) and D (same as C, except containing 1 M sodium chloride) buffers. Fractions containing FF A39G were concentrated and exchanged in the NMR buffer (100 mM sodium acetate, 200 mM sodium chloride, pH = 5.7) with Amicon Ultra 15 MWCO = 3 K (Merck, Darmstadt, Germany). The cleaved protein product was 71-residues long with no cloning artifacts at either end (GSQPAKKTYT WNTKEEAKQA FKELLKEKRV PSNASWEQGM KMIINDPRYS ALAKLSEKKQ AFNAYKVQTE K).

### NMR measurements

All NMR measurements were performed at 274.2 K using an AVANCE III HD 700 MHz spectrometer equipped with a TCI CryoProbe (Bruker BioSpin, Rheinstetten, Germany). Prior to recording the NMR measurements, the sample temperature was calibrated with methanol following the manufacturer’s instructions. The B_1_ field strength was calibrated with a ^15^N-labeled Tryptophan and Glutamine solution using a pulse program “hsqc_cest_f3gpphtc_b1cal” provided by the manufacturer. For the CEST measurements, 500 uL of 0.1 mM U-^13^C/^15^N FF A39G in the NMR buffer was loaded into a 5 mm NMR sample tube. Main-chain amide signals were sequentially assigned to standard triple resonance experiments [44–46]. To verify the accuracy of CEST-derived relaxation rate constants, ^15^N R_1_ and R_2_ were separately measured with the same sample using pulse programs “hsqct1etf3gpsi3d” and “hsqct2etf3gpsi,” respectively. The spectra were processed and analyzed using MATLAB 2020a (MathWorks, MA, USA). The detailed information for the NMR measurement is summarized in S15 Table.

### Adaptive CEST experiments

All CEST measurements were performed using a modified pulse program based on “hsqc_cest_etf3gpsitc3d”, which was originally designed for pseudo-3D measurement for ^1^H, ^15^N, and the irradiation-condition dimensions. We modified it to acquire a ^1^H-^15^N 2D plane under single irradiation conditions. Each 2D plane was acquired with 64 real points, 25-Hz spectral width, and 118.5-ppm carrier frequency in the ^15^N dimension, with eight cumulative transients corresponding to each time point. The adaptive NMR measurements were performed with the cooperation of two computers (Fig 6)—a control computer of the NMR system and a calculation computer (HP Z840 Workstation (HP Japan Inc., Tokyo, Japan) with dual Intel Xeon E5-2690v4 CPUs and 128 GiB main memory). On the control computer, an in-house C program running on TopSpin 3.5 software (Bruker BioSpin, Rheinstetten, Germany) was used to read the offset frequency, duration, and strength of each irradiation pulse from a local text file, start a measurement, and store raw time-domain data in local storage. On the calculation computer, another in-house program running on MATLAB 2020a (MathWorks, MA, USA) was used to write the irradiation conditions to the text file on the control computer, download the time-domain raw data, process them into spectra, analyze their peak intensities, perform MCMC for Bayesian inference of model parameters, and finally select each subsequent experimental condition based on mutual information. The in-house program utilized JSch 0.1.55 (JCraft, Sendai, Japan) for the SSH connection between the control and calculation computers, NMRglue 0.7 for NMR data processing [47], and mcmcstat for the MCMC analysis [48,49]. By the MCMC analysis, the following seven model parameters were inferred with either linear or logarithmic uniform prior distributions in the designated range: $p_{B}\in [0,0.1]$ (linear), $\begin{array}{c} k_{ex}\in [5,1000]{\text{s}}^{-1} \end{array}$ (logarithmic), $\begin{array}{c} \omega_{B}\in [-1000,1000]\text{H}z \end{array}$ (linear),$\begin{array}{c} R_{1}\in [0.1,10]{\text{s}}^{-1} \end{array}$ (logarithmic), $\frac{R_{2A}}{R_{1}}\in [1,100]$ (logarithmic), $\frac{R_{2B}}{R_{1}}\in [1,1000]$ (logarithmic), $\begin{array}{c} I_{0}\in [0.1,10000]\text{a}.\text{u}. \end{array}$ (logarithmic). The numbers of burn-in steps, sampling steps, and thinning intervals were 20,000, 30,000, and 50, respectively, in the mutual information calculation to reduce the computation time between NMR measurements. After all the iterations were completed, another iteration of MCMC with an increased number of steps (100,000 burn-in steps and 1,000,000 sampling steps) was performed for a more detailed analysis.

## Supporting information

S1 Text The theoretical background of the proposed method. (PDF)

S2 TextSecond-order approximation of $R_{1\rho }$ for calculation of the CEST forward model.(PDF)

S3 Table Computational time of various approximation methods. (PDF)

S4 Fig Approximation of the CEST forward model. (PDF)

S5 FigApproximation of the $R_{1\rho }$ relaxation constant.(PDF)

S6 Table The settings of CEST simulations in this study. (PDF)

S7 Table The true model parameters of the 1-signal virtual protein for simulations A1 and A2. (PDF)

S8 Fig The response to model-parameter changes in the simulation A1. (PDF)

S9 Fig Two-parameter joint and one-parameter marginal posterior distribution of the simulation A1. (PDF)

S10 Fig Model-parameter estimation of the simulation A2. (PDF)

S11 Table The true model parameters of the 70-signal virtual protein for simulations A3, A4, and C4. (PDF)

S12 Fig The model-parameter estimation of the adaptive and conventional CEST simulations. (PDF)

S13 Fig The model-parameter estimation for the representative residues of the adaptive CEST simulation A3. (PDF)

S14 Fig The estimated model parameters with or without additional iterations for the simulation A3. (PDF)

S15 Table Detailed information of the NMR measurements. (PDF)
