# Supplementary material for: Autonomous adaptive optimization of NMR experimental conditions for precise inference of minor conformational states of proteins based on chemical exchange saturation transfer
Source: PLoS One. 2025 May 16;20(5):e0321692. doi: 10.1371/journal.pone.0321692 (PMC12083826; doi:10.1371/journal.pone.0321692)
Supplement: S1 Text — (PDF) [file pone.0321692.s001.pdf]

S1 Text. The theoretical background of the proposed method.

Formularization of the CEST experiment

In this report, we varied three experimental parameters, $\boldsymbol{x} = \{\omega_{\mathrm{RF}}, \omega_1, T_{\mathrm{EX}}\}$, corresponding to the frequency, strength, and duration of the radio frequency irradiation pulse, respectively. In typical conventional 15N-CEST experiments, several tens of equally-spaced ω_{RF} are selected for each of a few different ω_1 while T_{EX} is fixed (1). In contrast, for $R_{1\rho}$ measurements, of which close relation to CEST was pointed out (2), several T_{EX} values are used to characterize $R_{1\rho}$ decay constants for a few different ω_{RF} and ω_1 (3). Therefore, the adaptive optimization of all of these experimental parameters should be regarded not only as adaptive CEST but also as adaptive $R_{1\rho}$ measurements. Although the parameters are intrinsically continuous, we select an experimental condition $\boldsymbol{x}$ from a discrete set $\boldsymbol{\mathcal{X}}$ in order to simplify the calculation. In adaptive CEST, we observe ${}^{(n)}\mathbf{Y} = \left\{{}^{(n)}y_k\right\}_{k=1}^{K} \in \mathbb{R}^K$ for each of the selected condition ${}^{(n)}\boldsymbol{x}$ at iteration n, where K is a number of signals and y_k is an intensity of the k-th signal. The iteration index (n) will be omitted unless necessary.

In this research, we assume two conformational states of the target molecule, the observable major state (A) and the invisible minor state (B). Then the model parameters to be estimated is $\mathbf{\Theta} = \{\boldsymbol{\theta}_k\}_{k=1}^{K}$, where $\boldsymbol{\theta}_k = \left\{p_{\mathrm{B}}^{(k)}, k_{\mathrm{ex}}^{(k)}, \omega_{\mathrm{B}}^{(k)}, R_1^{(k)}, R_{2\mathrm{A}}^{(k)}, R_{2\mathrm{B}}^{(k)}, I_0^{(k)}\right\}$ is the model parameters for the signal k. $p_{\mathrm{B}} = 1 - p_{\mathrm{A}}$ is the population ratio of the B state. k_{ex} is the exchange rate constant between both states. ω_{B} is the chemical shift of the B state. R_1 is the longitudinal relaxation rate constant of both states. Note that it is practical to assume $R_1 = R_{1\mathrm{A}} = R_{1\mathrm{B}}$ since CEST is insensitive to the differences in the longitudinal relaxation rates between both states (1). $R_{2\mathrm{A}}$ and $R_{2\mathrm{B}}$ are the transverse relaxation rate constants of the A and the B states, respectively. I_0 is the basal intensity at $T_{\mathrm{EX}} = 0$. The chemical shift of the A state, ω_{A}, is known from the observed spectrum. The non-linear fitting analysis using this type of modelling is referred as "local fitting" or "individual fitting" (1) since all model parameters are assumed to be independent of signals. Alternatively, "global fitting" assumes $\mathbf{\Theta} = \left\{\mathbf{\Gamma}, \{\boldsymbol{\theta}_k\}_{k=1}^{K}\right\}$, where $\mathbf{\Gamma} = \{p_{\mathrm{B}}, k_{\mathrm{ex}}\}$ and $\boldsymbol{\theta}_k = \left\{\omega_{\mathrm{B}}^{(k)}, R_1^{(k)}, R_{2\mathrm{A}}^{(k)}, R_{2\mathrm{B}}^{(k)}, I_0^{(k)}\right\}$, namely, p_{B} and k_{ex} are the same for all of the signals (1). This is expected in case of a structured single-domain protein with some exceptions due to intermolecular motions. Therefore it is practical to fit the data to the local model first in order to check the variances of p_{B} and k_{ex} and then switch to the global fitting if appropriate (1, 4).

The forward function and the observation model

For the two-state exchange system, the magnetization during the CEST irradiation pulse evolves under the Bloch-McConnell equation $\frac{d}{dt}\vec{M} = R\vec{M} + \vec{M}_{t=0}$ where $\vec{M} = \left(M_x^{\mathrm{A}}, M_y^{\mathrm{A}}, M_z^{\mathrm{A}}, M_x^{\mathrm{B}}, M_y^{\mathrm{B}}, M_z^{\mathrm{B}}\right)^T$ and

$$R = \begin{pmatrix} -R_2^{\mathrm{A}} - k_{\mathrm{AB}} & -\omega_{\mathrm{A}} & 0 & k_{\mathrm{BA}} & 0 & 0 \\ \omega_{\mathrm{A}} & -R_2^{\mathrm{A}} - k_{\mathrm{AB}} & -\omega_1 & 0 & k_{\mathrm{BA}} & 0 \\ 0 & \omega_1 & -R_1^{\mathrm{A}} - k_{\mathrm{AB}} & 0 & 0 & k_{\mathrm{BA}} \\ k_{\mathrm{AB}} & 0 & 0 & -R_2^{\mathrm{B}} - k_{\mathrm{BA}} & -\omega_{\mathrm{B}} & 0 \\ 0 & k_{\mathrm{AB}} & 0 & \omega_{\mathrm{B}} & -R_2^{\mathrm{B}} - k_{\mathrm{BA}} & -\omega_1 \\ 0 & 0 & k_{\mathrm{AB}} & 0 & \omega_1 & -R_1^{\mathrm{B}} - k_{\mathrm{BA}} \end{pmatrix}$$

(5). Therefore it is straightforward to analyze the CEST data by fitting them to a numerical solution of this differential equation (1). It should be noted that rapid decrease of the transverse magnetization in the tilted reference frame caused by B1 inhomogeneity should be additionally considered for this approach. In literature, the effect of B1 inhomogeneity was incorporated by summing result of different B1 fields (1) or by modifying a Liouvillian term (4, 6). Alternatively, the single exponential decay approximation was proposed because of its acceptable precision with faster computation time (2). Since this approach utilizes $R_{1\rho}$ approximation, we introduced a second-order approximation of $R_{1\rho}$, as described in S2 Text.

Let $f(\boldsymbol{x}, \boldsymbol{\theta})$ the adopted forward function. Since the thermal noise is dominant in the low SNR case, the observation noise is modelled by additive Gaussian:

$$y_k = f(\boldsymbol{x}, \boldsymbol{\theta}_k) + \varepsilon_k$$
$$\varepsilon_k \sim \mathcal{N}(0, {\sigma_k}^2)$$

Then the likelihood function is

$$p(y_k|\boldsymbol{\theta}_k) = \frac{1}{\sqrt{2\pi}\sigma_k} \exp\left(-\frac{\left(y_k - f(\boldsymbol{x}, \boldsymbol{\theta}_k)\right)^2}{2{\sigma_k}^2}\right)$$

Theoretically, Gaussian white noise added on the time-domain FID causes the same effect anywhere in the frequency-domain spectrum. However practically, ${\sigma_k}^2$ may vary between 15N-CEST signals due to the imperfectly suppressed water signal. In this study, ${\sigma_k}^2$ is assumed to be independent of the iteration index and estimated from the background noise of the observed spectra (see Experimental Section).

Bayesian analysis of the CEST experiments

For the CEST experiments, the model parameters are generally estimated by a non-linear fitting of the observed data to Bloch-McConnell equation or its approximation (1, 7). Recently, a neural network was used as an alternate to reduce computation time and to interpret complicated anti-phase CEST spectra, although so far the analyzable parameters are limited to chemical shifts (6). Instead of these methods, we employed Bayesian inference of the model parameters for three reasons. The first is that we require posterior distribution of the model parameters to calculate the utility function. The second reason is that Bayesian can infer arbitral posterior distribution. On the contrary, the estimation of the confidence intervals based on a linear approximation around the optimum may fail when the sample size is small (8), that is in case at the early stages of the sequential design. The third reason is that a prior distribution can be used to incorporate the prior knowledge of the system or to ensure model parameters to be evaluated fall into the region suitable for the approximation. In this work, we employed MCMC to sample from the arbitral posterior distribution.

Bayesian inference of the model parameters

After the measurement at iteration n, we infer the current knowledge about the model parameters of interest, $p(\boldsymbol{\Theta}|\boldsymbol{\mathcal{D}})$ using the available data $\boldsymbol{\mathcal{D}} = \left\{{}^{(i)}\mathbf{Y}\right\}_{i=1}^{n}$ by Bayesian theorem $p(\boldsymbol{\Theta}|\boldsymbol{\mathcal{D}}) \propto p(\boldsymbol{\mathcal{D}}|\boldsymbol{\Theta})p(\boldsymbol{\Theta})$. We adopted a continuous uniform distribution as the prior $p(\boldsymbol{\Theta})$ (see Experimental Section). We also assume $p(\boldsymbol{\Theta}) = \prod_{k=1}^{K} p(\boldsymbol{\theta}_k)$, i.e. the model parameters, including p_{B} and k_{ex}, of each residue are mutually independent. As described, it is likely that p_{B} and k_{ex} take the same value for all, or at least part of, signals especially in case of 15N-CEST experiment of a globular single-domain target protein. However, since it is not guaranteed, we should firstly assume they are independent, which is the similar procedure to the reported conventional CEST analyses (1, 4). Otherwise, experimental design based on improper prior knowledge without evidence may fail to pick up experimental conditions that have information to validate whether p_{B} and k_{ex} vary or not. It should be noted that after all data is corrected, multiple models, including the global model, can be tested as the final analysis using the whole data, regardless of which model was used for the experimental design.

Analytical posterior-distribution evaluation for the Bayesian experimental design of a non-linear

model is limited to simple models or depending on approximation (9). An alternate is sampling from the posterior including Markov-chain Monte Carlo (MCMC) and sequential Monte Carlo (SMC) or particle filter (9, 10, 11). The latter is reportedly advantageous because it is suitable to parallel computation and to updating nature of the sequential design (9, 12, 13). We employed MCMC instead because the assumption $p(\boldsymbol{\Theta}) = \prod_{k=1}^{K} p(\boldsymbol{\theta}_k)$ split the Bayesian inference computation on a signal-wise basis, $p(\boldsymbol{\theta}_k|\boldsymbol{\mathcal{D}}_k) \propto p(\boldsymbol{\mathcal{D}}_k|\boldsymbol{\theta}_k)p(\boldsymbol{\theta}_k)$, where $\boldsymbol{\mathcal{D}}_k = \left\{{}^{(i)}y_k\right\}_{i=1}^{n}$, which is inherently suitable for up to K-fold parallel computation.

The likelihood is calculated as:

$$p(\boldsymbol{\mathcal{D}}_k|\boldsymbol{\theta}_k) = \prod_{n=1}^{N} p\left({}^{(n)}y_k|\boldsymbol{\theta}_k\right) = \prod_{n=1}^{N} \frac{1}{\sqrt{2\pi}\sigma_k} \exp\left(-\frac{\left({}^{(n)}y_k - f(\boldsymbol{x}, \boldsymbol{\theta}_k)\right)^2}{2{\sigma_k}^2}\right)$$

Using MCMC sample $\left\{\boldsymbol{\theta}_k^{(s)}\right\}_{s=1}^{S}$ from $p(\boldsymbol{\theta}_k|\boldsymbol{\mathcal{D}}_k)$, an expectation over $p(\boldsymbol{\theta}_k|\boldsymbol{\mathcal{D}}_k)$ of an arbitrary function $G(\boldsymbol{\theta}_k)$ is approximated as:

$$\mathbb{E}_{\boldsymbol{\theta}_k|\boldsymbol{\mathcal{D}}_k}[G(\boldsymbol{\theta}_k)] = \int p(\boldsymbol{\theta}_k|\boldsymbol{\mathcal{D}}_k)G(\boldsymbol{\theta}_k)d\boldsymbol{\theta}_k \cong \frac{1}{S}\sum_{s=1}^{S} G\left(\boldsymbol{\theta}_k^{(s)}\right)$$

Bayesian experimental design

In the optimal experimental design, the experimental parameters are selected so that a utility function is maximized, which is defined depending on the purpose of the experiment. The expected gain of Shannon information was proposed for the utility function to the precise estimation of the model parameters (14, 15), which is equivalent to the expected Kullback-Leibler (KL) divergence and also to the mutual information (10, 16, 17). For the CEST experiment, to search a dip caused by the minor state is important (4, 18, 19, 20), which is unknown before the experiment. Therefore, adaptive optimization of the experimental condition by the sequential, or also called iterative, experimental design is required. After n-th iterations out of the total N iterations, a greedy strategy optimizing the very next iteration is guaranteed to lose only a small amount of information compared to a full intractable computation that considers all $N - n$ future observations (21).

Consider the design for the $n + 1$-th iteration after the n-th observation. Let $\hat{\mathbf{Y}}$ be the future

observation ${}^{(n+1)}\mathbf{Y}$. The prior distribution for the iteration is $p(\mathbf{\Theta}|\boldsymbol{\mathcal{D}})$, where $\boldsymbol{\mathcal{D}} = \left\{{}^{(i)}\mathbf{Y}\right\}_{i=1}^{n}$, which is estimated by MCMC after the n-th iteration. The posterior is $p\left(\mathbf{\Theta}|\boldsymbol{\mathcal{D}}, \widehat{\mathbf{Y}}\right)$. In this section, the prior and the posterior is written as $p(\mathbf{\Theta})$ and $p\left(\mathbf{\Theta}|\widehat{\mathbf{Y}}\right)$, respectively, by omitting $\boldsymbol{\mathcal{D}}$. The knowledge about the model parameters obtained in the $n+1$-th iteration is represented, for example, by the KL divergence of the posterior from the prior:

$$D_{\mathrm{KL}}\left(p\left(\mathbf{\Theta}|\widehat{\mathbf{Y}}\right)||p(\mathbf{\Theta})\right) = \int p\left(\mathbf{\Theta}|\widehat{\mathbf{Y}}\right) \log \frac{p\left(\mathbf{\Theta}|\widehat{\mathbf{Y}}\right)}{p(\mathbf{\Theta})} d\mathbf{\Theta}$$

As $\widehat{\mathbf{Y}}$ is the probabilistic future observation, the utility function $U(\boldsymbol{x})$ should be the expectation of the KL divergence over the current knowledge $p\left(\widehat{\mathbf{Y}}\right)$. This is equivalent to the mutual information $I\left(p\left(\widehat{\mathbf{Y}}\right); p(\mathbf{\Theta})\right)$. From the definition of the mutual information, two variables $\widehat{\mathbf{Y}}$ and $\mathbf{\Theta}$ can be swapped:

$$U(\boldsymbol{x}) = \mathbb{E}_{\widehat{\mathbf{Y}}}\left[D_{\mathrm{KL}}\left(p\left(\mathbf{\Theta}|\widehat{\mathbf{Y}}\right)||p(\mathbf{\Theta})\right)\right] = \iint p\left(\widehat{\mathbf{Y}}, \mathbf{\Theta}\right) \log \frac{p\left(\widehat{\mathbf{Y}}, \mathbf{\Theta}\right)}{p\left(\widehat{\mathbf{Y}}\right)p(\mathbf{\Theta})} d\widehat{\mathbf{Y}} d\mathbf{\Theta}$$

$$= I\left(p\left(\widehat{\mathbf{Y}}\right); p(\mathbf{\Theta})\right) = \mathbb{E}_{\mathbf{\Theta}}\left[\int p\left(\widehat{\mathbf{Y}}|\mathbf{\Theta}\right) \log \frac{p\left(\widehat{\mathbf{Y}}|\mathbf{\Theta}\right)}{p\left(\widehat{\mathbf{Y}}\right)} d\widehat{\mathbf{Y}}\right]$$

This converted form of $U(\boldsymbol{x})$ is easier to be computed (22) because the likelihood $p\left(\widehat{\mathbf{Y}}|\mathbf{\Theta}\right)$ can be analytically computed unlike the posterior $p\left(\mathbf{\Theta}|\widehat{\mathbf{Y}}\right)$ and the MCMC sample from the prior $p(\mathbf{\Theta})$ enables the approximation of the expectation $\mathbb{E}_{\mathbf{\Theta}}[\cdot]$. It is also pointed out that the dimensionality of the $\widehat{\mathbf{Y}}$ is usually low for the various experimental design problems so that the integration term is easy to be evaluated (22). This is not in our case, where $\widehat{\mathbf{Y}} = \{\hat{y}_k\}_{k=1}^{K} \in \mathbb{R}^K$. However, as discussed in the previous section, we assumed the mutual independence among $\boldsymbol{\theta}_k$ and consequently $\hat{y}_k$ which leads to

$$I\left(p\left(\widehat{\mathbf{Y}}\right); p(\mathbf{\Theta})\right) = \iint p\left(\widehat{\mathbf{Y}}, \mathbf{\Theta}\right) \log \frac{p\left(\widehat{\mathbf{Y}}, \mathbf{\Theta}\right)}{p\left(\widehat{\mathbf{Y}}\right)p(\mathbf{\Theta})} d\widehat{\mathbf{Y}} d\mathbf{\Theta}$$

$$= \iint \prod_{j=1}^{K} p(\hat{y}_j, \boldsymbol{\theta}_j) \sum_{k=1}^{K} \log \frac{p(\hat{y}_k, \boldsymbol{\theta}_k)}{p(\hat{y}_k)p(\boldsymbol{\theta}_k)} d\widehat{\mathbf{Y}} d\mathbf{\Theta}$$

$$= \sum_{k=1}^{K} \left[\iint p(\hat{y}_k, \boldsymbol{\theta}_k) \log \frac{p(\hat{y}_k, \boldsymbol{\theta}_k)}{p(\hat{y}_k)p(\boldsymbol{\theta}_k)} d\hat{y}_k d\boldsymbol{\theta}_k\right.$$

$$\left.\times \iint \prod_{j \neq k} p(\hat{y}_j, \boldsymbol{\theta}_j)\, d\widehat{\mathbf{Y}}_{\backslash k} d\mathbf{\Theta}_{\backslash k}\right] = \sum_{k=1}^{K} I(p(\hat{y}_k); p(\boldsymbol{\theta}_k))$$

In short, the total mutual information is the sum of the signal-wise mutual information.

Since $p(\hat{y}_k) = \int p(\hat{y}_k|\boldsymbol{\theta}_k)p(\boldsymbol{\theta}_k)d\boldsymbol{\theta}_k = \mathbb{E}_{\boldsymbol{\theta}_k}[p(\hat{y}_k|\boldsymbol{\theta}_k)]$, the signal-wise mutual information can be written as:

$$I(p(\hat{y}_k); p(\boldsymbol{\theta}_k)) = \mathbb{E}_{\boldsymbol{\theta}_k}\left[\int p(\hat{y}_k|\boldsymbol{\theta}_k)\{\log p(\hat{y}_k|\boldsymbol{\theta}_k) - \log \mathbb{E}_{\boldsymbol{\theta}_k}[p(\hat{y}_k|\boldsymbol{\theta}_k)]\}d\hat{y}_k\right]$$

Approximation of the both of $\mathbb{E}_{\boldsymbol{\theta}_k}[\cdot]$ with the MCMC sample yields

$$I(p(\hat{y}_k); p(\boldsymbol{\theta}_k)) \simeq \frac{1}{S}\sum_{s=1}^{S}\int p\left(\hat{y}_k|\boldsymbol{\theta}_k^{(s)}\right)\left\{\log p\left(\hat{y}_k|\boldsymbol{\theta}_k^{(s)}\right) - \log\frac{1}{S}\sum_{t=1}^{S} p\left(\hat{y}_k|\boldsymbol{\theta}_k^{(t)}\right)\right\}d\hat{y}_k$$

The evaluation of the term requires integral along $\hat{y}_k$. For Bayesian experimental design of non-linear models, the utility functions are evaluated along of the observation $\hat{\mathbf{Y}}$ in various way (9, 10) including MCMC or SMC sampling in the space spanned by both $\boldsymbol{\Theta}$ and $\hat{\mathbf{Y}}$ (23), importance sampling along $\hat{\mathbf{Y}}$ (24, 25), and combining individual two MCMCs along $\boldsymbol{\Theta}$ and $\hat{\mathbf{Y}}$ (26). To reduce computational time, we employed Riemann sum approximation within a range $[\min_{s=1\ldots S} f\left(\boldsymbol{x}, \boldsymbol{\theta}_k^{(s)}\right) - 4\sigma_k, \max_{s=1\ldots S} f\left(\boldsymbol{x}, \boldsymbol{\theta}_k^{(s)}\right) + 4\sigma_k]$ with a step σ_k. It should be noted that $\min_{s=1\ldots S} f\left(\boldsymbol{x}, \boldsymbol{\theta}_k^{(s)}\right) = 0$ since the forward function $f(\boldsymbol{x}, \boldsymbol{\theta})$ is always non-negative and is equal to 0 if $\omega_{\mathrm{RF}} = \omega_{\mathrm{A}}$ and $T_{\mathrm{EX}} > 0$.

After evaluation of $U(\boldsymbol{x})$ for all $\boldsymbol{x} \in \boldsymbol{X}$, we select the next experimental condition ${}^{(n+1)}\boldsymbol{x}$ by

$${}^{(n+1)}\boldsymbol{x} = \operatorname*{argmax}_{x \in \boldsymbol{X}} U(\boldsymbol{x})$$

allowing repetition, i.e. the same condition may be selected at multiple iterations. In case low SNR, the repetition may be important because some uncertainty of $f(\hat{\boldsymbol{x}}, \boldsymbol{\theta}_k)$ remains after a single observation $\hat{y}_k = f(\hat{\boldsymbol{x}}, \boldsymbol{\theta}_k) + \varepsilon_k$ and should be reduced by the sampling of $\varepsilon_k \sim \mathcal{N}(0, {\sigma_k}^2)$. On the other hand, for example in the extreme high SNR case, $\sigma_k = 0$, remaining no uncertainty leads to $U(\boldsymbol{x}) = 0$ for $\boldsymbol{x}$ which is already selected.

References

1. Vallurupalli P, Bouvignies G, Kay LE. Studying “Invisible” Excited Protein States in Slow Exchange with a Major State Conformation. Journal of the American Chemical Society. 2012;134(19):8148-61.

2. Palmer AG. Chemical exchange in biomacromolecules: Past, present, and future. Journal of Magnetic Resonance. 2014;241:3-17.

3. Massi F, Johnson E, Wang C, Rance M, Palmer AG. NMR $R_{1\rho}$ Rotating-Frame

Relaxation with Weak Radio Frequency Fields. Journal of the American Chemical Society. 2004;126(7):2247-56.
4. Bolik-Coulon N, Hansen DF, Kay LE. Optimizing frequency sampling in CEST experiments. Journal of Biomolecular NMR. 2022;76(5):167-83.
5. McConnell HM. Reaction Rates by Nuclear Magnetic Resonance. The Journal of Chemical Physics. 1958;28(3):430-1.
6. Karunanithy G, Yuwen T, Kay LE, Hansen DF. Towards autonomous analysis of chemical exchange saturation transfer experiments using deep neural networks. Journal of Biomolecular NMR. 2022;76(3):75-86.
7. Vallurupalli P, Sekhar A, Yuwen T, Kay LE. Probing conformational dynamics in biomolecules via chemical exchange saturation transfer: a primer. Journal of Biomolecular NMR. 2017;67(4):243-71.
8. Lee SY. Bayesian Nonlinear Models for Repeated Measurement Data: An Overview, Implementation, and Applications. Mathematics. 2022;10(6):898.
9. Ryan EG, Drovandi CC, McGree JM, Pettitt AN. A Review of Modern Computational Algorithms for Bayesian Optimal Design. International Statistical Review. 2016;84(1):128-54.
10. Chaloner K, Verdinelli I. Bayesian Experimental Design: A Review. Statistical Science. 1995;10(3):273-304, 32.
11. Jiang H, Zhao Y. A Review of Bayesian Optimal Experimental Design on Different Models. In: Zhao Y, Chen D-G, editors. Modern Statistical Methods for Health Research. Cham: Springer International Publishing; 2021. p. 205-20.
12. Amzal B, Bois FY, Parent E, Robert CP. Bayesian-Optimal Design via Interacting Particle Systems. Journal of the American Statistical Association. 2006;101(474):773-85.
13. Cavagnaro DR, Myung JI, Pitt MA, Kujala JV. Adaptive Design Optimization: A Mutual Information-Based Approach to Model Discrimination in Cognitive Science. Neural Computation. 2010;22(4):887-905.
14. Lindley DV. On a Measure of the Information Provided by an Experiment. The Annals of Mathematical Statistics. 1956;27(4):986-1005, 20.
15. Shannon CE. A mathematical theory of communication. The Bell System Technical Journal. 1948;27(3):379-423.
16. Cover TM, Thomas JA. Elements of Information Theory. Hoboken, NJ: John Wiley & Sons, Inc.; 1991.
17. Kullback S, Leibler RA. On Information and Sufficiency. The Annals of Mathematical Statistics. 1951;22(1):79-86, 8.
18. Leninger M, Marsiglia WM, Jerschow A, Traaseth NJ. Multiple frequency saturation pulses reduce CEST acquisition time for quantifying conformational exchange in biomolecules.

Journal of Biomolecular NMR. 2018;71(1):19-30.
19. Yuwen T, Bouvignies G, Kay LE. Exploring methods to expedite the recording of CEST datasets using selective pulse excitation. Journal of Magnetic Resonance. 2018;292:1-7.
20. Yuwen T, Kay LE, Bouvignies G. Dramatic Decrease in CEST Measurement Times Using Multi-Site Excitation. ChemPhysChem. 2018;19(14):1707-10.
21. Golovin D, Krause A. Adaptive submodularity: theory and applications in active learning and stochastic optimization. J Artif Int Res. 2011;42(1):427–86.
22. Houlsby N, Huszár F, Ghahramani Z, Lengyel M. Bayesian Active Learning for Classification and Preference Learning2011 December 01, 2011:[arXiv:1112.5745 p.]. Available from: https://ui.adsabs.harvard.edu/abs/2011arXiv1112.5745H.
23. van Den Berg J, Curtis A, Trampert J. Optimal nonlinear Bayesian experimental design: an application to amplitude versus offset experiments. Geophysical Journal International. 2003;155(2):411-21.
24. Ryan KJ. Estimating Expected Information Gains for Experimental Designs With Application to the Random Fatigue-Limit Model. Journal of Computational and Graphical Statistics. 2003;12(3):585-603.
25. Drovandi CC, McGree JM, Pettitt AN. Sequential Monte Carlo for Bayesian sequentially designed experiments for discrete data. Computational Statistics & Data Analysis. 2013;57(1):320-35.
26. Vanlier J, Tiemann CA, Hilbers PAJ, van Riel NAW. A Bayesian approach to targeted experiment design. Bioinformatics. 2012;28(8):1136-42.
