# Supplementary material for: Autonomous adaptive optimization of NMR experimental conditions for precise inference of minor conformational states of proteins based on chemical exchange saturation transfer
Source: PLoS One. 2025 May 16;20(5):e0321692. doi: 10.1371/journal.pone.0321692 (PMC12083826; doi:10.1371/journal.pone.0321692)
Supplement: S2 Text — (PDF) [file pone.0321692.s002.pdf]

S2 Text. Second-order approximation of $R_{1\rho}$ for calculation of the CEST forward model.

Along with the full calculation of Bloch-McConnell equation for CEST analysis (1), the following single exponential decay approximation is acceptable alternate with faster computation time (2):

$$I(T_{\mathrm{EX}}) = \cos^2\psi \exp(-R_{1\rho}T_{\mathrm{EX}})\, I_0$$

$$\cos^2\psi = (\omega_{\mathrm{A}} - \omega_{\mathrm{RF}})^2/({\omega_1}^2 + (\omega_{\mathrm{A}} - \omega_{\mathrm{RF}})^2)$$

where $I(t)$ is the signal intensity at time t, ψ is the tilt angle of the reference frame, and $R_{1\rho}$ is a longitudinal relaxation constant in the tilted reference frame. The propagation matrix of Bloch-McConnell equation of the two-state model is

$$R = \begin{pmatrix} -R_2^{\mathrm{A}} - k_{\mathrm{AB}} & -\omega_{\mathrm{A}} & 0 & k_{\mathrm{BA}} & 0 & 0 \\ \omega_{\mathrm{A}} & -R_2^{\mathrm{A}} - k_{\mathrm{AB}} & -\omega_1 & 0 & k_{\mathrm{BA}} & 0 \\ 0 & \omega_1 & -R_1^{\mathrm{A}} - k_{\mathrm{AB}} & 0 & 0 & k_{\mathrm{BA}} \\ k_{\mathrm{AB}} & 0 & 0 & -R_2^{\mathrm{B}} - k_{\mathrm{BA}} & -\omega_{\mathrm{B}} & 0 \\ 0 & k_{\mathrm{AB}} & 0 & \omega_{\mathrm{B}} & -R_2^{\mathrm{B}} - k_{\mathrm{BA}} & -\omega_1 \\ 0 & 0 & k_{\mathrm{AB}} & 0 & \omega_1 & -R_1^{\mathrm{B}} - k_{\mathrm{BA}} \end{pmatrix}$$

The Bloch-McConnell equation is $\frac{d}{dt}\vec{M} = R\vec{M} + \vec{M}_{t=0}$ where $\vec{M} = \left(M_x^{\mathrm{A}}, M_y^{\mathrm{A}}, M_z^{\mathrm{A}}, M_x^{\mathrm{B}}, M_y^{\mathrm{B}}, M_z^{\mathrm{B}}\right)^T$. $R_{1\rho}$ is the smallest real eigenvalue in magnitude of R and therefore it can be obtained by solving the following characteristic equation which is 6-order polynomial with respect to λ (3, 4):

$$R_{1\rho} = -\lambda$$

$$\det|R - \lambda E| = 0 \Leftrightarrow C_0 + C_1\lambda + C_2\lambda^2 + \cdots + C_6\lambda^6 = 0$$

For most of practical $R_{1\rho}$ measurements, we can assume that $R_{1\rho}$ is small because otherwise the decay is too fast to be observed (3, 4). For CEST experiments, although $R_{1\rho}$ may become large in case of on-resonance irradiation, a misevaluation of $R_{1\rho}$ in such large $R_{1\rho}$ region merely affects the final observation, I/I_0. Therefore, if the final I/I_0 calculated with an $R_{1\rho}$ approximation method which assuming small $R_{1\rho}$ is sufficiently accurate, such $R_{1\rho}$ approximation is helpful for the CEST analysis.

Consequently we approximate the equation either to the first or the second order by assuming $R_{1\rho}$ is small as follows:

$$\lambda \approx -\frac{C_0}{C_1}$$

$$\lambda \approx \frac{-C_1 + \sqrt{{C_1}^2 - 4C_0C_2}}{2C_2}$$

To calculate C_0, C_1, and C_2, we introduce the following new notations to further simplify the propagation matrix R:

$$K_2^{\mathrm{A}} = R_2^{\mathrm{A}} + k_{\mathrm{AB}}, \qquad K_1^{\mathrm{A}} = R_1^{\mathrm{A}} + k_{\mathrm{AB}}$$
$$K_2^{\mathrm{B}} = R_2^{\mathrm{B}} + k_{\mathrm{BA}}, \qquad K_1^{\mathrm{B}} = R_1^{\mathrm{B}} + k_{\mathrm{BA}}$$

$$R = \begin{pmatrix} -K_2^{\mathrm{A}} & -\omega_{\mathrm{A}} & 0 & k_{\mathrm{BA}} & 0 & 0 \\ \omega_{\mathrm{A}} & -K_2^{\mathrm{A}} & -\omega_1 & 0 & k_{\mathrm{BA}} & 0 \\ 0 & \omega_1 & -K_1^{\mathrm{A}} & 0 & 0 & k_{\mathrm{BA}} \\ k_{\mathrm{AB}} & 0 & 0 & -K_2^{\mathrm{B}} & -\omega_{\mathrm{B}} & 0 \\ 0 & k_{\mathrm{AB}} & 0 & \omega_{\mathrm{B}} & -K_2^{\mathrm{B}} & -\omega_1 \\ 0 & 0 & k_{\mathrm{AB}} & 0 & \omega_1 & -K_1^{\mathrm{B}} \end{pmatrix}$$

Using this notation, C_0, C_1, and C_2, consists of 25, 42, and 39 terms, respectively. Instead of calculating all terms separately, the following conversions are made to reduce the computational costs:

$$k = k_{\mathrm{AB}} k_{\mathrm{BA}}$$
$$D_{\mathrm{A}} = {\omega_{\mathrm{A}}}^2, \qquad D_{\mathrm{B}} = {\omega_{\mathrm{B}}}^2, \qquad D = \omega_{\mathrm{A}}\omega_{\mathrm{B}}$$
$$K_1 = K_1^{\mathrm{A}} K_1^{\mathrm{B}}, \qquad K_2 = K_2^{\mathrm{A}} K_2^{\mathrm{B}}, \qquad K_{\mathrm{A}} = K_1^{\mathrm{A}} K_2^{\mathrm{A}}, \qquad K_{\mathrm{B}} = K_1^{\mathrm{B}} K_2^{\mathrm{B}}$$
$$K_{\mathrm{AB}} = K_1^{\mathrm{A}} K_2^{\mathrm{B}}, \qquad K_{\mathrm{BA}} = K_1^{\mathrm{B}} K_2^{\mathrm{A}}$$
$$L = K_{\mathrm{AB}} + K_{\mathrm{BA}}, \qquad L_1 = K_1^{\mathrm{A}} + K_1^{\mathrm{B}}, \qquad L_2 = K_2^{\mathrm{A}} + K_2^{\mathrm{B}}$$

$$S_1 = {K_2}^2 + {K_2^{\mathrm{A}}}^2 D_{\mathrm{B}} + {K_2^{\mathrm{B}}}^2 D_{\mathrm{A}} + D^2$$
$$S_2 = 2K_{\mathrm{A}} + 2K_{\mathrm{B}}$$
$$S_3 = 2K_1 + K_2 + 2L + S_2$$
$$S_4 = {K_2^{\mathrm{A}}}^2 + {K_2^{\mathrm{B}}}^2$$
$$S_5 = D_{\mathrm{A}} + D_{\mathrm{B}} + S_4 + 4K_2$$
$$S_6 = D - K_2$$

$$C_{04} = K_2 - k$$

$$C_{02} = -2k^2 - k(K_{\mathrm{A}} + K_{\mathrm{B}} + 2S_6) + K_{\mathrm{AB}}\left(D_{\mathrm{A}} + {K_2^{\mathrm{A}}}^2\right) + K_{\mathrm{BA}}\left(D_{\mathrm{B}} + {K_2^{\mathrm{B}}}^2\right)$$

$$C_{00} = k^2(K_1 - 2S_6 - k) + k(2K_1S_6 - S_1) + K_1S_1$$
$$C_{14} = L_2$$
$$C_{12} = k(L_2 - L_1) + K_2L_2 + K_{\mathrm{A}}\left(L_2 + K_2^{\mathrm{A}}\right) + K_{\mathrm{B}}\left(L_2 + K_2^{\mathrm{B}}\right) + D_{\mathrm{A}}\left(K_1^{\mathrm{A}} + K_2^{\mathrm{B}}\right) + D_{\mathrm{B}}\left(K_1^{\mathrm{B}} + K_2^{\mathrm{A}}\right)$$

$$C_{10} = k^2(2L_2 - L_1) + 2k\{-K_2^{\mathrm{A}}D_{\mathrm{B}} - K_2^{\mathrm{B}}D_{\mathrm{A}} + L_1(S_6 - K_1 - K_2)\}$$
$$+ D_{\mathrm{A}}\left({K_2^{\mathrm{B}}}^2 L_1 + 2K_2^{\mathrm{B}}K_1\right) + D_{\mathrm{B}}\left({K_2^{\mathrm{A}}}^2 L_1 + 2K_2^{\mathrm{A}}K_1\right) + D^2L_1$$
$$+ K_2(K_2L_1 + 2K_1L_2)$$
$$C_{24} = 1$$
$$C_{22} = L + S_2 + S_5$$
$$C_{20} = 3k^2 + k(D - S_3 - S_5 + S_6) + D_{\mathrm{A}}\left({K_2^{\mathrm{B}}}^2 + 2K_{\mathrm{AB}} + 2K_{\mathrm{B}} + K_1\right)$$
$$+ D_{\mathrm{B}}\left({K_2^{\mathrm{A}}}^2 + 2K_{\mathrm{BA}} + 2K_{\mathrm{A}} + K_1\right) + D^2 + K_2(2K_1 + S_3) + K_1S_4$$

Finally, we obtain:

$$C_0 = C_{04}{\omega_1}^4 + C_{02}{\omega_1}^2 + C_{00}$$
$$C_1 = C_{14}{\omega_1}^4 + C_{12}{\omega_1}^2 + C_{10}$$
$$C_2 = C_{24}{\omega_1}^4 + C_{22}{\omega_1}^2 + C_{20}$$

In terms of I/I_0, the accuracy of the second-order approximation of was comparable to $R_{1\rho}$ calculation by eigenvalue (S4 Figure). As it is accurate all over the parameter region which may be visited in MCMC, it helped stability of the posterior evaluation by MCMC (S4 Figure). The computation was 30-fold faster than numerical eigenvalue calculation, while it is acceptably slower by 24–28% compared with the other first-order based $R_{1\rho}$ approximation methods (S3 Table).

It should be noted that the second-order approximation is more accurate than the other first-order approximation methods also in terms of $R_{1\rho}$, especially in case $R_{1\rho}$ is small (S5 Figure). Therefore, if the increase of the computation time is acceptable (S3 Table), it supersedes the first-order approximations for the analysis of $R_{1\rho}$ relaxation measurements.

References

1. McConnell HM. Reaction Rates by Nuclear Magnetic Resonance. The Journal of Chemical Physics. 1958;28(3):430-1.
2. Palmer AG. Chemical exchange in biomacromolecules: Past, present, and future. Journal of Magnetic Resonance. 2014;241:3-17.
3. Baldwin AJ, Kay LE. An $R_{1\rho}$ expression for a spin in chemical exchange between two sites with unequal transverse relaxation rates. Journal of Biomolecular NMR. 2013;55(2):211-8.
4. Trott O, Palmer AG. $R_{1\rho}$ Relaxation outside of the Fast-Exchange Limit. Journal of Magnetic Resonance. 2002;154(1):157-60.
