# Supplementary material for: Autonomous adaptive optimization of NMR experimental conditions for precise inference of minor conformational states of proteins based on chemical exchange saturation transfer
Source: PLoS One. 2025 May 16;20(5):e0321692. doi: 10.1371/journal.pone.0321692 (PMC12083826; doi:10.1371/journal.pone.0321692)
Supplement: S3 Table — (PDF) [file pone.0321692.s003.pdf]

S3 Table. Computational time of various approximation methods[a].

	CEST [μs]	$R_{1\rho}$ [μs]
Bloch-McConnell	$(4.72\pm0.02)\times10^6$	not applicable
Eigenvalue	883±6	923±3
Trott & Palmer	23.6±0.1	11.0±0.1
Baldwin & Kay	24.4±0.1	11.9±0.2
This work (first order)	24.2±0.2	10.9±0.1
This work (second order)	30.2±0.2	14.0±0.2

[a]As a single functional call, either I/I_0 (of the CEST experiment) or $R_{1\rho}$ was calculated for 100 randomly generated experimental conditions against a randomly generated model parameter on a single physical CPU core of Xeon E5-2690 v4 2.6 GHz (Intel), which emulated a typical forward-model evaluation situation at the MCMC step. A single run contained 8–1,048,576 functional calls with different random seeds so that the total computational time of the run were within 10–40 s. The computational time of the single functional call, the total time divided by the number of functional calls per run, was presented followed by the standard deviation of 11 runs.
