# Supplementary material for: Autonomous adaptive optimization of NMR experimental conditions for precise inference of minor conformational states of proteins based on chemical exchange saturation transfer
Source: PLoS One. 2025 May 16;20(5):e0321692. doi: 10.1371/journal.pone.0321692 (PMC12083826; doi:10.1371/journal.pone.0321692)
Supplement: S4 Fig — (PDF) [file pone.0321692.s004.pdf]

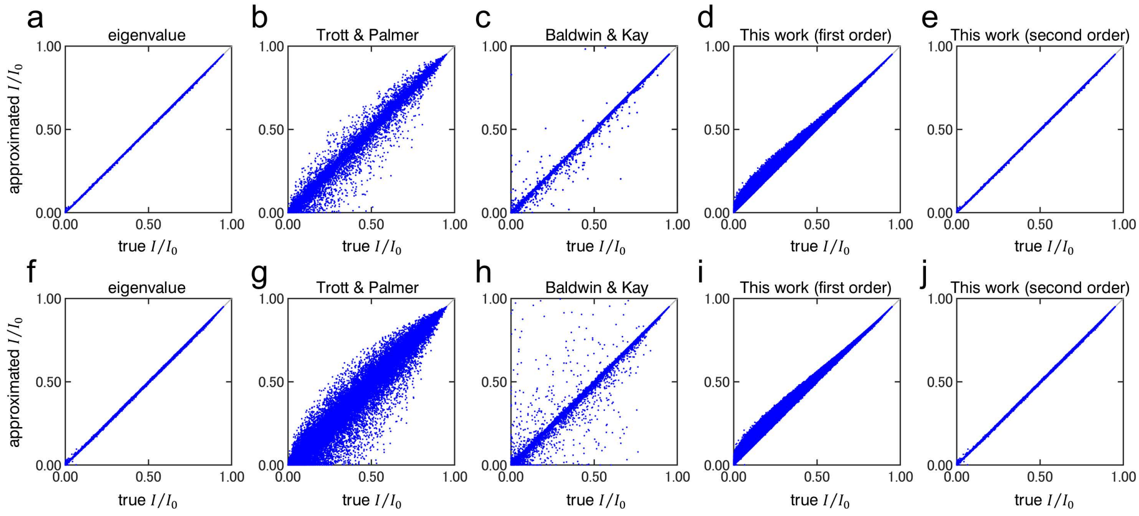

S4 Figure. Approximation of the CEST forward model.

I/I_0 of the CEST forward model using Palmer's approximation (1) combined with various $R_{1\rho}$ calculation methods were plotted against true I/I_0 by the numerical integration of Bloch-McConnell equation. (a) $R_{1\rho}$ calculation by eigenvalue. (b) $R_{1\rho}$ approximation by Trott and Palmer (2). (c) $R_{1\rho}$ approximation by Baldwin and Kay (3). (d) The first-order $R_{1\rho}$ approximation of the presented work. (e) The second-order $R_{1\rho}$ approximation of the presented work. I/I_0 values were plotted for randomly generated 100,000 parameters within the MCMC parameter region used in the presented work, i.e.,$-1{,}000\ \mathrm{Hz} \le \omega_\mathrm{A}, \omega_\mathrm{B}, \omega_\mathrm{RF} \le 1{,}000\ \mathrm{Hz},\ 0 \le p_\mathrm{B} \le 0.1,\ 5\ \mathrm{s}^{-1} \le k_\mathrm{ex} \le 1{,}000\ \mathrm{s}^{-1}$,

$0.1\ \mathrm{s}^{-1} \le R_1 \le 10\ \mathrm{s}^{-1}, 1 \le R_{2\mathrm{A}}/R_1 \le 100, 1 \le R_{2\mathrm{B}}/R_1 \le 1{,}000, 5\ \mathrm{Hz} \le \omega_1 \le 100\ \mathrm{Hz}$,

$0.5\ \mathrm{s} \le \mathrm{T_{EX}} \le 1.0\ \mathrm{s}$.

(f-j) The same as (a-e) except for $-5{,}000\ \mathrm{Hz} \le \omega_\mathrm{A}, \omega_\mathrm{B}, \omega_\mathrm{RF} \le 5{,}000\ \mathrm{Hz}$. I/I_0 values were plotted for randomly generated 500,000 parameters

References

1. Palmer AG. Chemical exchange in biomacromolecules: Past, present, and future. Journal of Magnetic Resonance. 2014;241:3-17.

2. Trott O, Palmer AG. $R_{1\rho}$ Relaxation outside of the Fast-Exchange Limit. Journal of Magnetic Resonance. 2002;154(1):157-60.

3. Baldwin AJ, Kay LE. An $\mathrm{R}_{1\rho}$ expression for a spin in chemical exchange between two sites with unequal transverse relaxation rates. Journal of Biomolecular NMR. 2013;55(2):211-8.
