# Supplementary material for: Autonomous adaptive optimization of NMR experimental conditions for precise inference of minor conformational states of proteins based on chemical exchange saturation transfer
Source: PLoS One. 2025 May 16;20(5):e0321692. doi: 10.1371/journal.pone.0321692 (PMC12083826; doi:10.1371/journal.pone.0321692)
Supplement: S5 Fig — (PDF) [file pone.0321692.s005.pdf]

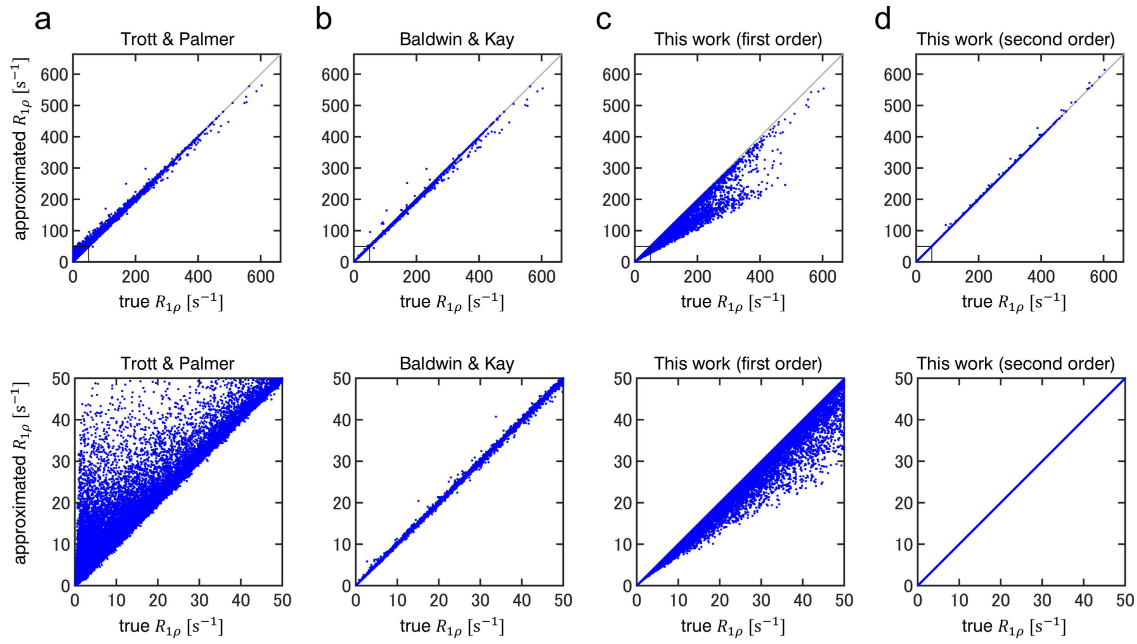

S5 Figure. Approximation of the $R_{1\rho}$ relaxation constant.

Approximated $R_{1\rho}$ were plotted against true $R_{1\rho}$ by numerical eigenvalue calculation. Along with the full-region plots (top), the expanded small $R_{1\rho}$ areas were presented (bottom). (b) $R_{1\rho}$ approximation by Trott and Palmer (1). (c) $R_{1\rho}$ approximation by Baldwin and Kay (2). (d) The first-order $R_{1\rho}$ approximation of the presented work. (e) The second-order $R_{1\rho}$ approximation of the presented work. $R_{1\rho}$ values were plotted for randomly generated 100,000 parameters within a region according to Baldwin and Kay (2), i.e., $\omega_{\mathrm{RF}} = 0, 0.001\ \mathrm{rad/s} \leq \omega_{\mathrm{A}} \leq 100{,}000\ \mathrm{rad/s}, 12.5\ \mathrm{rad/s} \leq |\omega_{\mathrm{B}} - \omega_{\mathrm{A}}| \leq 12{,}500\ \mathrm{rad/s},\ 0.001 \leq p_{\mathrm{B}} \leq 0.1,\ 10\ \mathrm{s}^{-1} \leq k_{\mathrm{ex}} \leq 20{,}000\ \mathrm{s}^{-1},\ 0.1\ \mathrm{s}^{-1} \leq R_1 \leq 10\ \mathrm{s}^{-1}, 0.1\ \mathrm{s}^{-1} \leq R_{2\mathrm{A}} \leq R_{2\mathrm{B}} \leq 500\ \mathrm{s}^{-1}, 25\ \mathrm{rad/s} \leq \omega_1 \leq 100{,}000\ \mathrm{rad/s}, \sqrt{{\omega_{\mathrm{A}}}^2 + {\omega_1}^2} > 0.1\mathrm{s/rad} \times \left(p_{\mathrm{B}} \times (\omega_{\mathrm{B}} - \omega_{\mathrm{A}})\right)^2$.

References

1. Trott O, Palmer AG. $R_{1\rho}$ Relaxation outside of the Fast-Exchange Limit. Journal of Magnetic Resonance. 2002;154(1):157-60.

2. Baldwin AJ, Kay LE. An $\mathrm{R}_{1\rho}$ expression for a spin in chemical exchange between two sites with unequal transverse relaxation rates. Journal of Biomolecular NMR. 2013;55(2):211-8.
