# Supplementary material for: Autonomous adaptive optimization of NMR experimental conditions for precise inference of minor conformational states of proteins based on chemical exchange saturation transfer
Source: PLoS One. 2025 May 16;20(5):e0321692. doi: 10.1371/journal.pone.0321692 (PMC12083826; doi:10.1371/journal.pone.0321692)
Supplement: S6 Table — (PDF) [file pone.0321692.s006.pdf]

S6 Table. The settings of CEST simulations in this study.

Type	Adaptive				Conventional
Simulation number	A1	A2	A3	A4	C4
Number of signals	1	1	70	70	70
ω_{RF}	401 points, 5 Hz step, [-1000, 1000] Hz	401 points, 5 Hz step, [-1000, 1000] Hz	401 points, 5 Hz step, [-1000, 1000] Hz	401 points, 5 Hz step, [-1000, 1000] Hz	63 points, 32.3 Hz step, [-1000, 1000] Hz
ω_1 [Hz]	0, 10, or 50	0 or 15	0, 6.3, 13.0, 26.2 or 50.0	0, 6.3, 13.0 or 26.2	0, 6.3, 13.0 or 26.2
T_{EX} [s]	0 or 0.5	0, 0.50, 0.75, or 1.00	0, 0.5, or 1.0	0, 0.5, or 1.0	0 or 0.5
Number of experimental condition candidates	803	1204	3211	2409	192
Number of iterations	200	200	192	192	n/a
Number of 2D measurements	200	200	192	192	192
The background noise σ of the simulated 2D spectra	0.59	0.59	0.59	0.59	0.59

n/a: not applicable.

S6 Table (continued.)

Type	Conventional				
Simulation number	C5	C6	C7	C8	C9
Number of signals	70	70	70	70	70
ω_{RF}	127 points, 15.9 Hz step for $\omega_1 =$ 6.3 Hz), 63 points, 32.3 Hz step for $\omega_1 =$ 13.0 Hz) , [-1000, 1000] Hz	127 points, 15.9 Hz step for $\omega_1 =$ 13.0 Hz), 63 points, 32.3 Hz step for $\omega_1 =$ 26.2 Hz) , [-1000, 1000] Hz	191 points, 10.5 Hz step, [-1000, 1000] Hz	191 points, 10.5 Hz step, [-1000, 1000] Hz	191 points, 10.5 Hz step, [-1000, 1000] Hz
ω_1 [Hz]	0, 6.3, or 13.0	0, 13.0, or 26.2	0 or 6.3	0 or 13.0	0 or 26.2
T_{EX} [s]	0 or 0.5	0 or 0.5	0 or 0.5	0 or 0.5	0 or 0.5
Number of experimental condition candidates	192	192	192	192	192
Number of iterations	n/a	n/a	n/a	n/a	n/a
Number of 2D measurements	192	192	192	192	192
The background noise σ of the simulated 2D spectra	0.59	0.59	0.59	0.59	0.59

n/a: not applicable.

S6 Table (continued.)

Type	Conventional	
Simulation number	C10	C11
Number of signals	70	70
ω_{RF}	127 points, 4.0 Hz step for $\omega_1 =$ 13.0 Hz), 63 points, 8.1 Hz step for $\omega_1 =$ 26.2 Hz) , [0, 500] Hz	191 points, 2.6 Hz step, [0, 500] Hz
ω_1 [Hz]	0, 13.0, or 26.2	0 or 13.0
T_{EX} [s]	0 or 0.5	0 or 0.5
Number of experimental condition candidates	192	192
Number of iterations	n/a	n/a
Number of 2D measurements	192	192
The background noise σ of the simulated 2D spectra	0.59	0.59

n/a: not applicable.
