# Supplementary material for: Autonomous adaptive optimization of NMR experimental conditions for precise inference of minor conformational states of proteins based on chemical exchange saturation transfer
Source: PLoS One. 2025 May 16;20(5):e0321692. doi: 10.1371/journal.pone.0321692 (PMC12083826; doi:10.1371/journal.pone.0321692)
Supplement: S7 Table — (PDF) [file pone.0321692.s007.pdf]

S7 Table. The true model parameters of the 1-signal virtual protein for simulations A1 and A2.

Residue Name	p_B	k_{ex} [s^{-1}]	ω_A [Hz]	ω_B [Hz]	R_1 [s^{-1}]	R_{2A} [s^{-1}]	R_{2B} [s^{-1}]	I_0 [a.u.]
X	0.0150	50.0	-300.0	300.0	1.000	15.00	100.00	11.8
