# Supplementary material for: Autonomous adaptive optimization of NMR experimental conditions for precise inference of minor conformational states of proteins based on chemical exchange saturation transfer
Source: PLoS One. 2025 May 16;20(5):e0321692. doi: 10.1371/journal.pone.0321692 (PMC12083826; doi:10.1371/journal.pone.0321692)
Supplement: S8 Fig — (PDF) [file pone.0321692.s008.pdf]

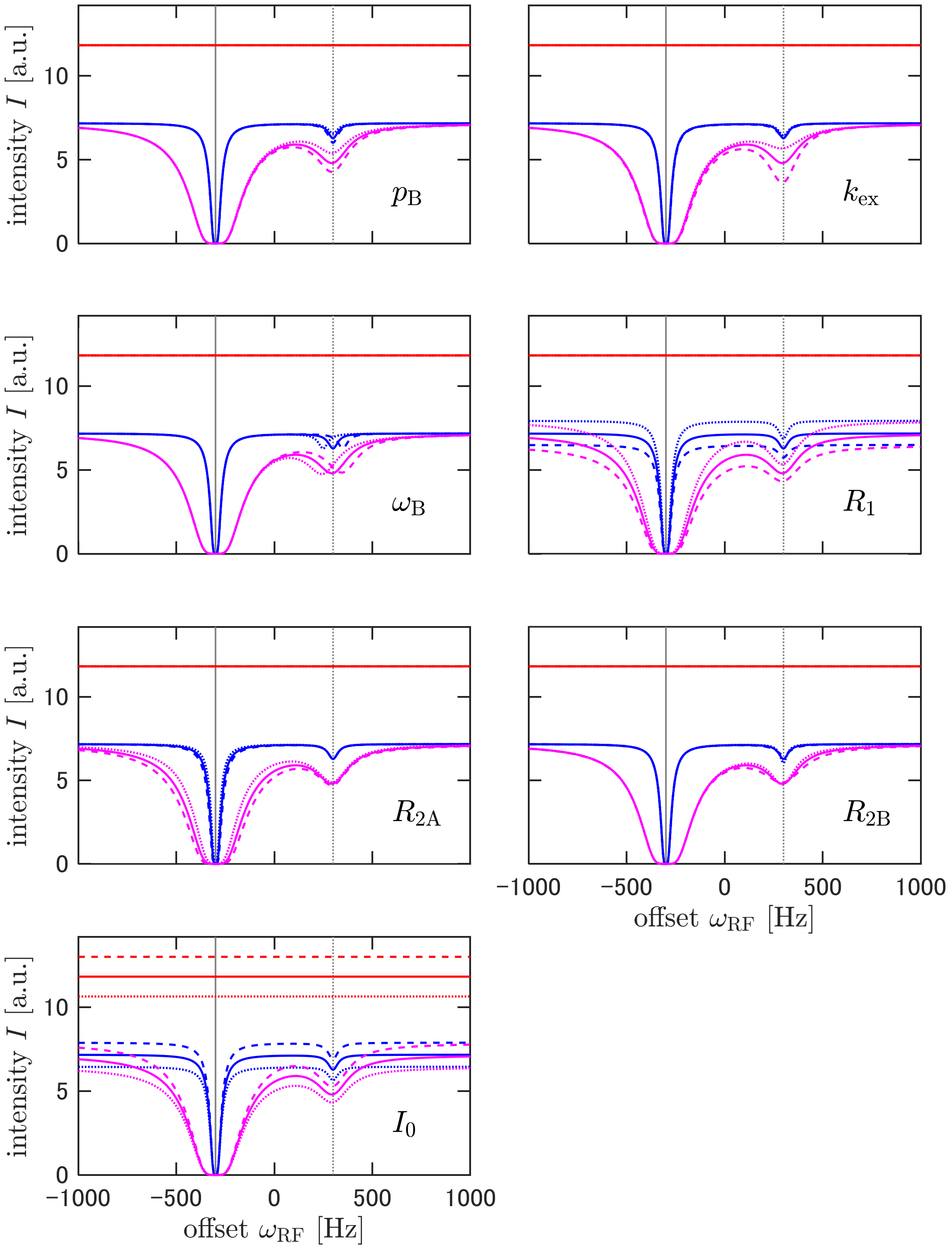

S8 Figure. The response to model-parameter changes in the simulation A1. Solid lines are generated by the forward function with the true model parameter ($p_B = 1.5\%$, $k_{ex} = 50\ s^{-1}$, $\omega_A = -300$ Hz, $\omega_B = 300$ Hz, $R_1 = 1\ s^{-1}$, $R_{2A} = 15\ s^{-1}$, $R_{2B} = 100\ s^{-1}$, and $I_0 = 11.8$ a.u.). In each panel, the designated model parameter is increased (dashed lines) or decreased (dotted lines). Red, blue, and magenta colors correspond to the different ω_1, 0, 10, and 50 Hz, respectively. Vertical solid and dotted gray lines indicate the true ω_A and ω_B, respectively. (a)

$p_{\mathrm{B}} = 2.0\%$ (dashed) or 1.0% (dotted), (b) $k_{\mathrm{ex}} = 100\ \mathrm{s}^{-1}$ (dashed) or $25\ \mathrm{s}^{-1}$ (dotted), (c) $\omega_{\mathrm{B}} = 350\ \mathrm{Hz}$ (dashed) or $250\ \mathrm{Hz}$ (dotted), (d) $R_1 = 1.2\ \mathrm{s}^{-1}$ (dashed) or $0.8\ \mathrm{s}^{-1}$ (dotted), (e) $R_{2\mathrm{A}} = 20\ \mathrm{s}^{-1}$ (dashed) or $10\ \mathrm{s}^{-1}$ (dotted), (f) $R_{2\mathrm{B}} = 200\ \mathrm{s}^{-1}$ (dashed) or $50\ \mathrm{s}^{-1}$ (dotted), and (g) $I_0 = 13.0$ a.u. (dashed) or 10.6 a.u. (dotted).
