# Supplementary material for: Autonomous adaptive optimization of NMR experimental conditions for precise inference of minor conformational states of proteins based on chemical exchange saturation transfer
Source: PLoS One. 2025 May 16;20(5):e0321692. doi: 10.1371/journal.pone.0321692 (PMC12083826; doi:10.1371/journal.pone.0321692)
Supplement: S9 Fig — (PDF) [file pone.0321692.s009.pdf]

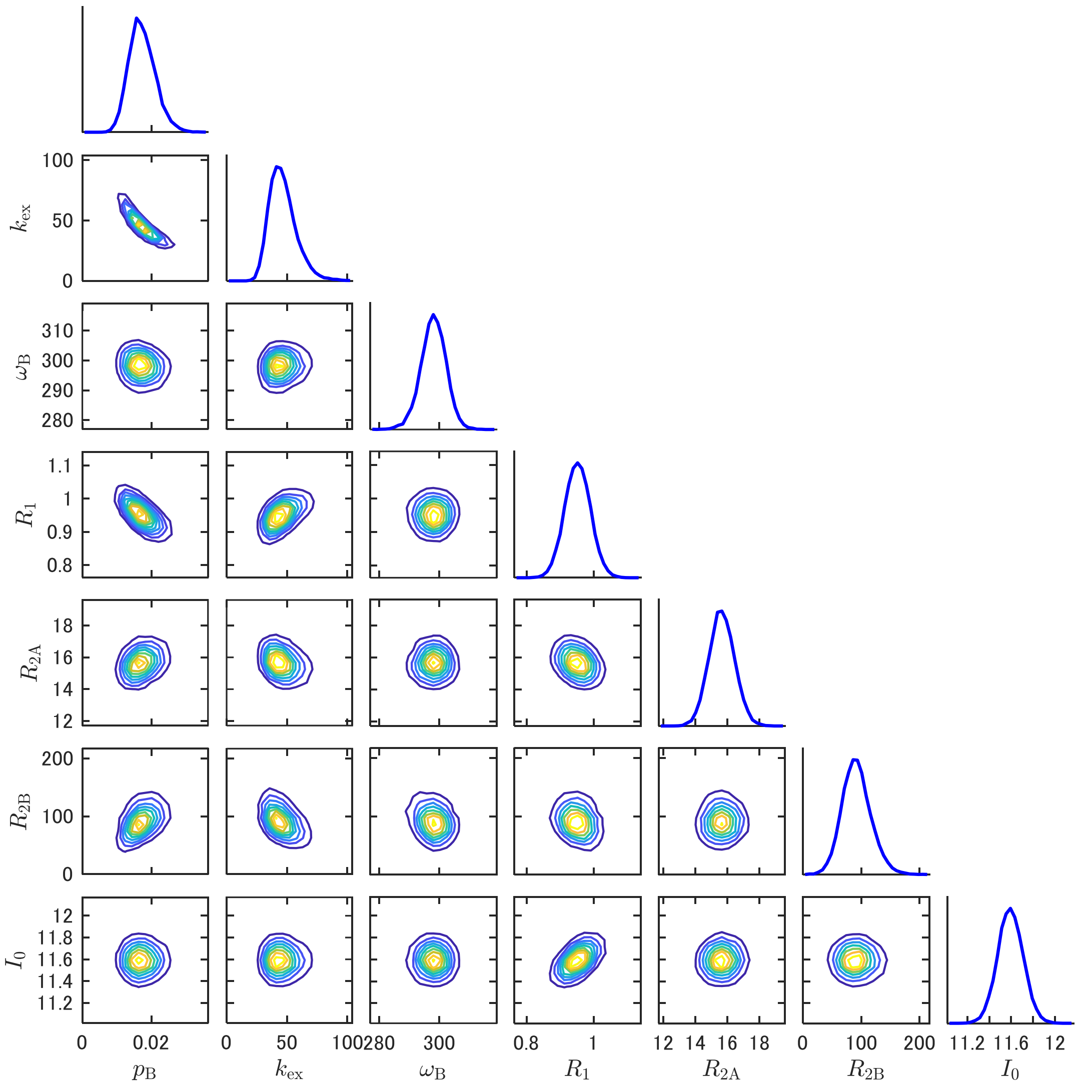

S9 Figure. Two-parameter joint and one-parameter marginal posterior distribution of the simulation A1.
