# Supplementary material for: Autonomous adaptive optimization of NMR experimental conditions for precise inference of minor conformational states of proteins based on chemical exchange saturation transfer
Source: PLoS One. 2025 May 16;20(5):e0321692. doi: 10.1371/journal.pone.0321692 (PMC12083826; doi:10.1371/journal.pone.0321692)
Supplement: S11 Table — (PDF) [file pone.0321692.s011.pdf]

S11 Table. The true model parameters of the 70-signal virtual protein for simulations A3, A4, and C4.

Residue Name	p_B	k_{ex} [s^{-1}]	ω_A [Hz]	ω_B [Hz]	R_1 [s^{-1}]	R_{2A} [s^{-1}]	R_{2B} [s^{-1}]	I_0 [a.u.]
X2	0.0201	47.3	-211.1	-160.7	0.829	1.46	0.84	50.6
X3	0.0208	50.4	252.0	144.4	1.118	1.51	1.13	62.9
X4	0.0214	47.5	317.7	242.4	1.247	2.37	1.26	72.6
X5	0.0211	44.3	134.2	245.2	1.430	3.48	1.44	61.9
X6	0.0212	50.2	275.3	281.5	1.409	4.13	1.42	61.7
X7	0.0200	44.7	167.7	285.1	1.379	6.14	1.39	50.5
X8	0.0215	48.8	42.1	-184.7	1.279	6.93	1.29	33.9
X9	0.0213	49.2	-36.7	266.9	1.111	9.91	17.32	26.8
X10	0.0223	46.4	209.4	61.7	1.008	12.54	33.05	19.8
X11	0.0228	45.4	190.2	-12.6	0.927	14.60	72.97	14.4
X12	0.0215	46.0	591.8	171.5	0.967	16.32	192.41	17.1
X13	0.0193	44.9	128.3	237.1	0.963	18.55	159.85	19.3
X14	0.0203	46.5	18.2	25.8	0.931	14.85	140.16	25.3
X15	0.0205	45.4	340.1	174.8	0.989	13.13	70.07	19.6
X16	0.0209	42.4	354.7	278.2	0.897	14.99	53.02	20.4
X17	0.0208	49.8	226.5	353.1	0.961	11.97	42.24	10.7
X18	0.0228	51.3	-0.4	152.6	0.948	12.97	52.97	9.6
X19	0.0192	45.7	-296.0	193.6	0.938	12.44	65.51	16.2
X20	0.0224	45.1	464.8	406.7	0.954	14.12	86.28	13.2
X21	0.0216	45.1	97.4	319.6	0.961	14.30	101.90	20.7
X22	0.0217	52.5	-420.2	-379.0	1.004	14.53	164.00	16.0
X23	0.0216	49.9	-1.3	113.6	0.933	13.40	89.54	14.7
X24	0.0213	48.1	485.7	97.1	0.959	14.32	125.95	19.9
X25	0.0211	46.3	365.7	306.0	0.964	12.76	93.40	14.6
X26	0.0180	50.9	87.5	206.1	0.945	13.41	161.44	17.7
X27	0.0205	47.3	354.1	167.8	0.948	13.41	158.19	14.4
X28	0.0211	49.8	532.3	426.8	0.966	13.11	218.84	13.6
X29	0.0219	48.5	260.6	243.2	0.938	12.67	467.64	16.4
X30	0.0210	47.9	57.6	192.8	0.954	13.93	113.33	21.1
X31	0.0210	45.2	-543.1	-444.4	0.962	14.29	123.67	27.6
X32	0.0202	54.0	414.8	296.1	0.951	14.06	140.38	30.8

X33	0.0203	45.3	348.8	334.2	0.940	13.97	29.11	19.8
X34	0.0217	43.5	-11.5	6.7	0.949	12.54	19.87	20.0
X35	0.0198	47.3	492.7	325.6	0.909	12.09	78.47	22.6
X36	0.0220	47.1	290.5	367.1	0.958	13.70	147.13	14.9
X37	0.0203	45.5	144.3	217.4	0.974	15.50	202.51	17.2
X38	0.0205	49.6	361.7	207.8	0.929	11.61	213.77	19.0
X39	0.0211	46.9	102.7	314.7	0.933	13.45	99.49	16.1
X40	0.0217	49.1	237.7	146.4	0.931	13.48	123.87	13.1
X41	0.0209	51.8	284.4	240.6	0.925	13.66	188.20	19.5
X42	0.0205	44.1	470.3	269.1	0.958	12.37	244.49	22.4
X43	0.0210	49.2	129.3	83.2	0.946	12.92	332.74	18.6
X44	0.0211	52.3	58.5	116.1	0.936	12.52	273.37	13.5
X45	0.0219	48.9	144.7	33.6	0.933	14.96	279.15	17.8
X46	0.0218	49.4	129.8	72.1	0.930	14.95	178.49	15.1
X47	0.0201	47.3	517.8	307.6	0.956	13.43	166.96	16.5
X48	0.0189	49.7	290.1	147.7	0.941	13.75	79.35	20.6
X49	0.0212	49.7	-33.4	175.0	0.942	12.24	37.06	13.7
X50	0.0220	46.8	152.4	258.8	0.943	13.70	33.07	20.4
X51	0.0181	50.0	-595.0	-674.3	0.966	13.24	19.37	13.7
X52	0.0218	42.8	-392.4	211.1	0.939	15.65	28.20	17.5
X53	0.0219	52.0	381.5	144.2	0.937	14.52	46.23	12.5
X54	0.0209	45.4	319.2	314.2	0.952	14.25	59.12	19.6
X55	0.0218	47.5	584.4	271.2	0.931	13.16	140.19	19.8
X56	0.0217	46.8	120.5	-12.7	0.941	13.65	102.83	21.2
X57	0.0204	46.3	110.8	235.0	0.935	17.57	193.79	23.2
X58	0.0223	54.1	185.6	196.7	0.953	15.48	209.88	19.4
X59	0.0223	46.5	245.0	256.9	0.968	13.74	231.97	14.2
X60	0.0216	48.2	174.9	84.1	0.941	14.17	85.71	18.2
X61	0.0219	47.9	717.3	311.6	0.960	13.91	104.93	12.7
X62	0.0213	45.9	655.7	295.2	0.943	13.49	140.39	18.6
X63	0.0205	44.7	328.8	226.2	0.961	13.39	112.58	18.0
X64	0.0214	42.6	409.7	263.5	0.933	13.81	149.60	16.7
X65	0.0217	48.0	21.4	185.7	0.942	14.62	202.79	19.3
X66	0.0208	45.6	158.3	186.4	0.958	18.09	176.80	19.4
X67	0.0214	45.9	-85.8	-18.8	1.000	14.10	42.83	16.8

X68	0.0204	42.7	56.5	24.2	1.168	12.24	24.75	30.6
X69	0.0217	50.9	184.0	229.4	1.222	8.29	9.40	32.1
X70	0.0204	43.4	139.7	201.7	1.295	7.98	1.31	44.6
X71	0.0217	54.3	99.9	144.7	1.379	5.49	1.39	45.5
