# Supplementary material for: Autonomous adaptive optimization of NMR experimental conditions for precise inference of minor conformational states of proteins based on chemical exchange saturation transfer
Source: PLoS One. 2025 May 16;20(5):e0321692. doi: 10.1371/journal.pone.0321692 (PMC12083826; doi:10.1371/journal.pone.0321692)
Supplement: S12 Fig — (PDF) [file pone.0321692.s012.pdf]

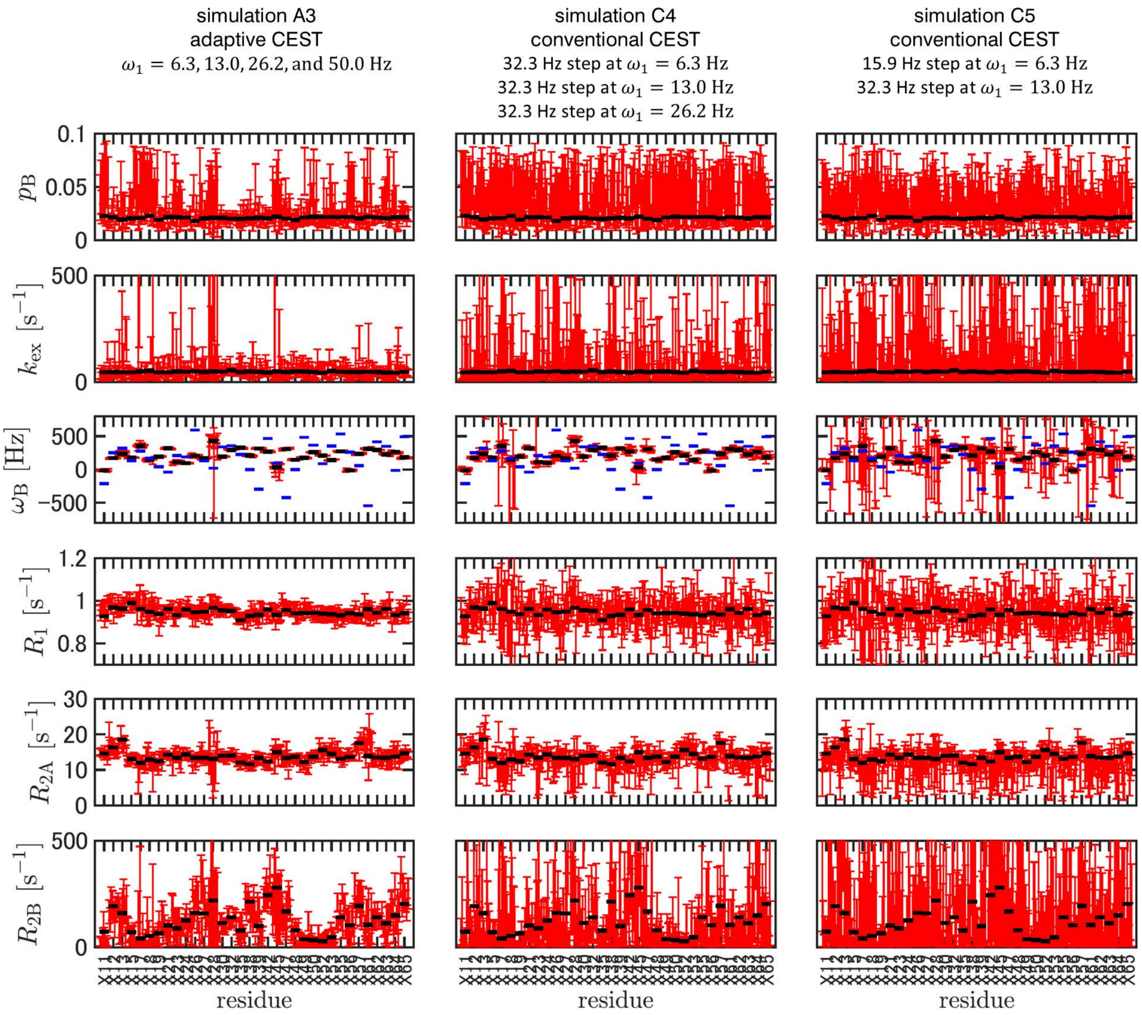
simulation A3
adaptive CEST
ω_1 = 6.3, 13.0, 26.2, and 50.0 Hz
simulation C4
conventional CEST
32.3 Hz step at ω_1 = 6.3 Hz
32.3 Hz step at ω_1 = 13.0 Hz
32.3 Hz step at ω_1 = 26.2 Hz
simulation C5
conventional CEST
15.9 Hz step at ω_1 = 6.3 Hz
32.3 Hz step at ω_1 = 13.0 Hz
p_B
k_{ex} [s^{-1}]
ω_B [Hz]
R_1 [s^{-1}]
R_{2A} [s^{-1}]
R_{2B} [s^{-1}]
residue

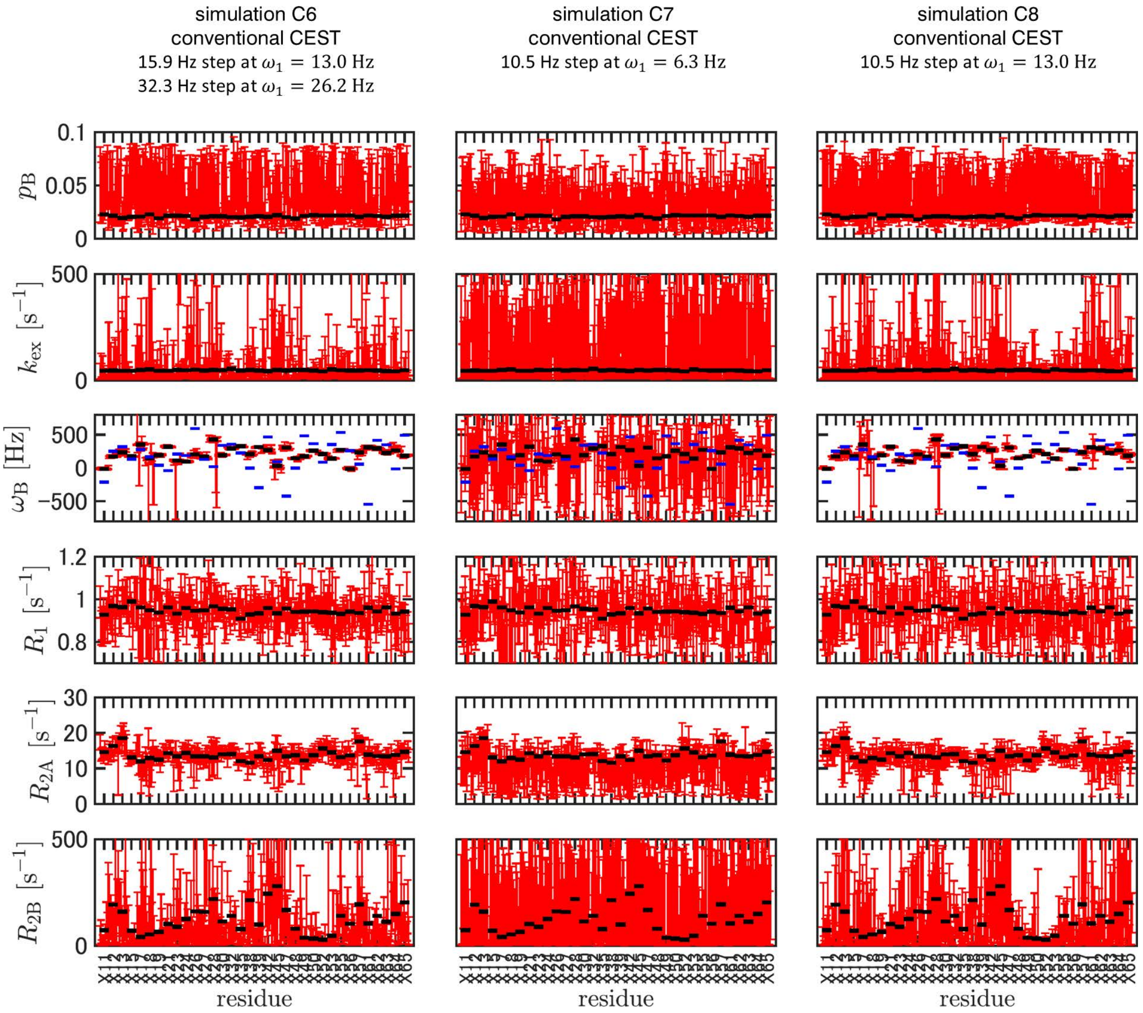
simulation C6
conventional CEST
15.9 Hz step at ω_1 = 13.0 Hz
32.3 Hz step at ω_1 = 26.2 Hz
simulation C7
conventional CEST
10.5 Hz step at ω_1 = 6.3 Hz
simulation C8
conventional CEST
10.5 Hz step at ω_1 = 13.0 Hz
p_B
k_{ex} [s^{-1}]
ω_B [Hz]
R_1 [s^{-1}]
R_{2A} [s^{-1}]
R_{2B} [s^{-1}]
residue
residue
residue

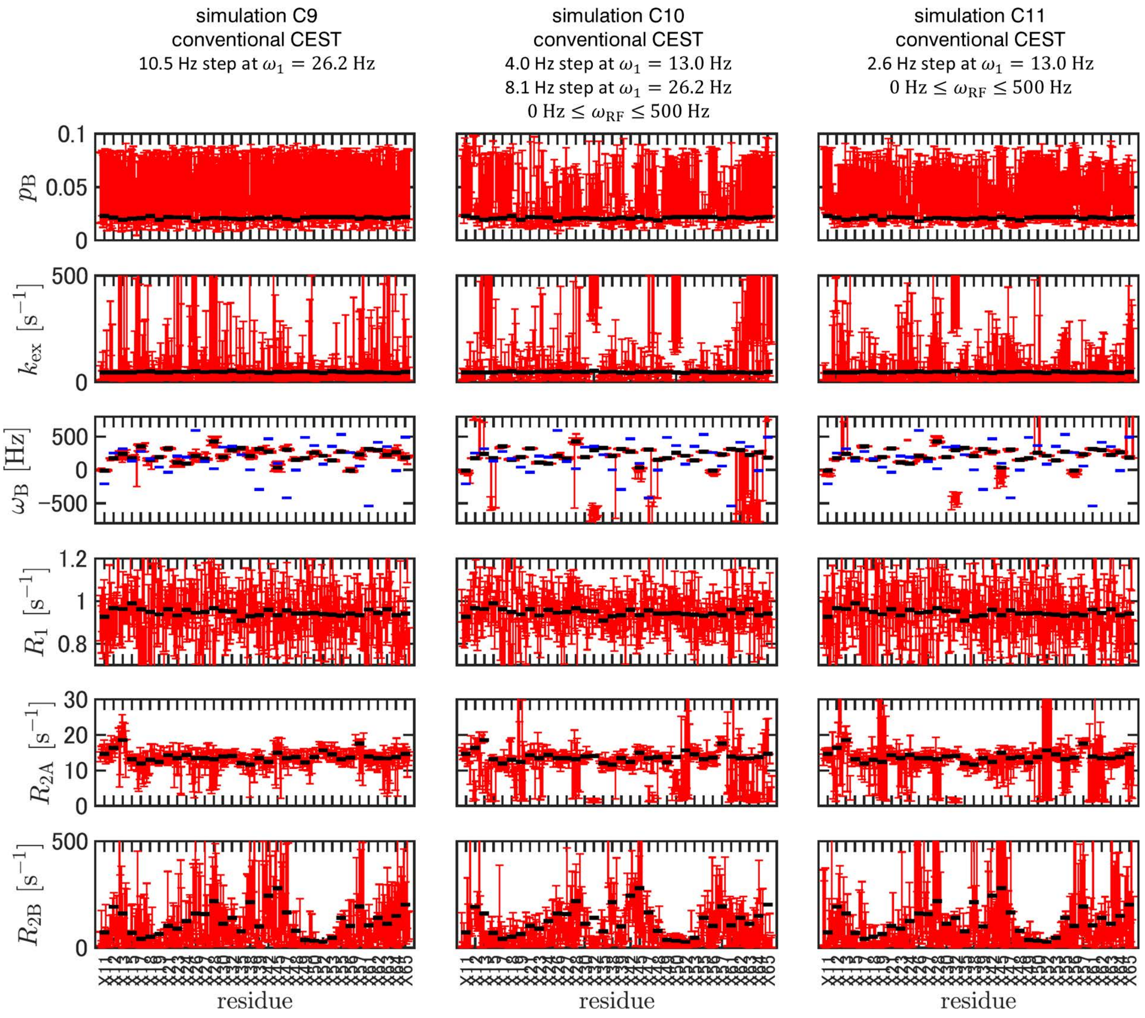

S12 Figure. The model-parameter estimation of the adaptive and conventional CEST simulations. All simulations were performed with 192 2D measurements. From left to right; adaptive CEST simulation A3 with $\omega_1 = 6.3,\ 13.0,\ 26.2,$ and 50.0 Hz (reproduced from Fig 4a left for comparison); conventional CEST simulation C4 with 32.3 Hz step 63 measurements at $\omega_1 = 6.3,\ 13.0,$ and 26.2 Hz (reproduced from Fig 4a right for comparison); conventional CEST simulation C5 with 15.9 Hz step 127 measurements at $\omega_1 = 6.3$ Hz and 32.3 Hz step 63 measurements at $\omega_1 = 13.0$ Hz; conventional CEST simulation C6 with 15.9 Hz step 127 measurements at $\omega_1 = 13.0$ Hz and 32.3 Hz step 63 measurements at $\omega_1 = 26.2$ Hz; conventional CEST simulation C7 with 10.5 Hz step 191 measurements at $\omega_1 = 6.3$ Hz; conventional CEST simulation C8 with 10.5 Hz step 191 measurements at $\omega_1 = 13.0$ Hz; conventional CEST simulation C9 with 10.5 Hz step 191 measurements at $\omega_1 = 26.2$ Hz; conventional CEST simulation C10 with 4.0 Hz step 127 measurements at $\omega_1 = 13.0$ Hz and 8.1 Hz step 63 measurements at $\omega_1 = 26.2$ Hz, $0\ \text{Hz} \le \omega_{RF} \le 500$ Hz; conventional CEST simulation C11 with 2.6 Hz step 191 measurements at $\omega_1 = 13.0$ Hz, $0\ \text{Hz} \le \omega_{RF} \le 500$ Hz.
