# Supplementary material for: Autonomous adaptive optimization of NMR experimental conditions for precise inference of minor conformational states of proteins based on chemical exchange saturation transfer
Source: PLoS One. 2025 May 16;20(5):e0321692. doi: 10.1371/journal.pone.0321692 (PMC12083826; doi:10.1371/journal.pone.0321692)
Supplement: S13 Fig — (PDF) [file pone.0321692.s013.pdf]

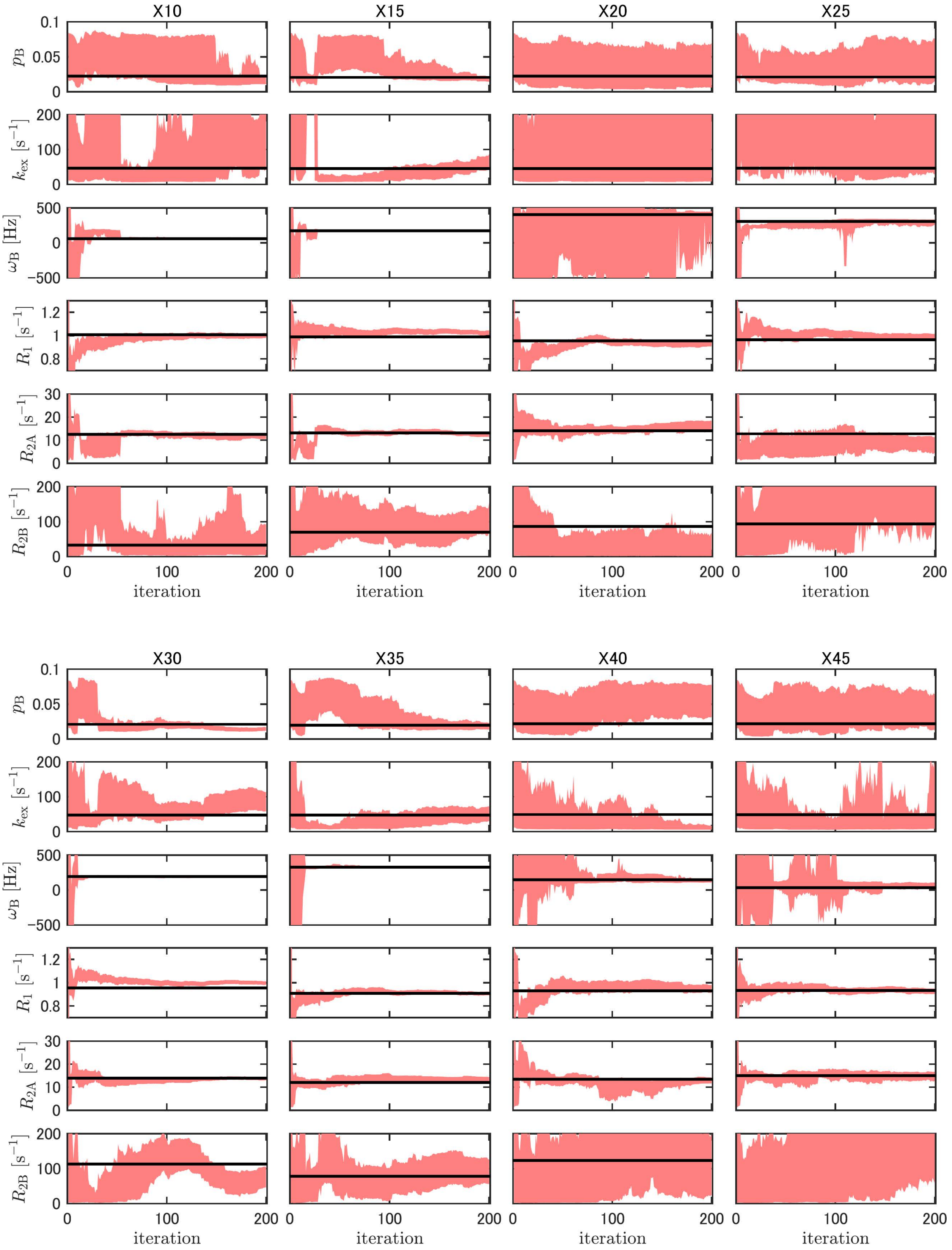

X10
X15
X20
X25
X30
X35
X40
X45
p_B
k_{ex} [s^{-1}]
ω_B [Hz]
R_1 [s^{-1}]
R_{2A} [s^{-1}]
R_{2B} [s^{-1}]
iteration

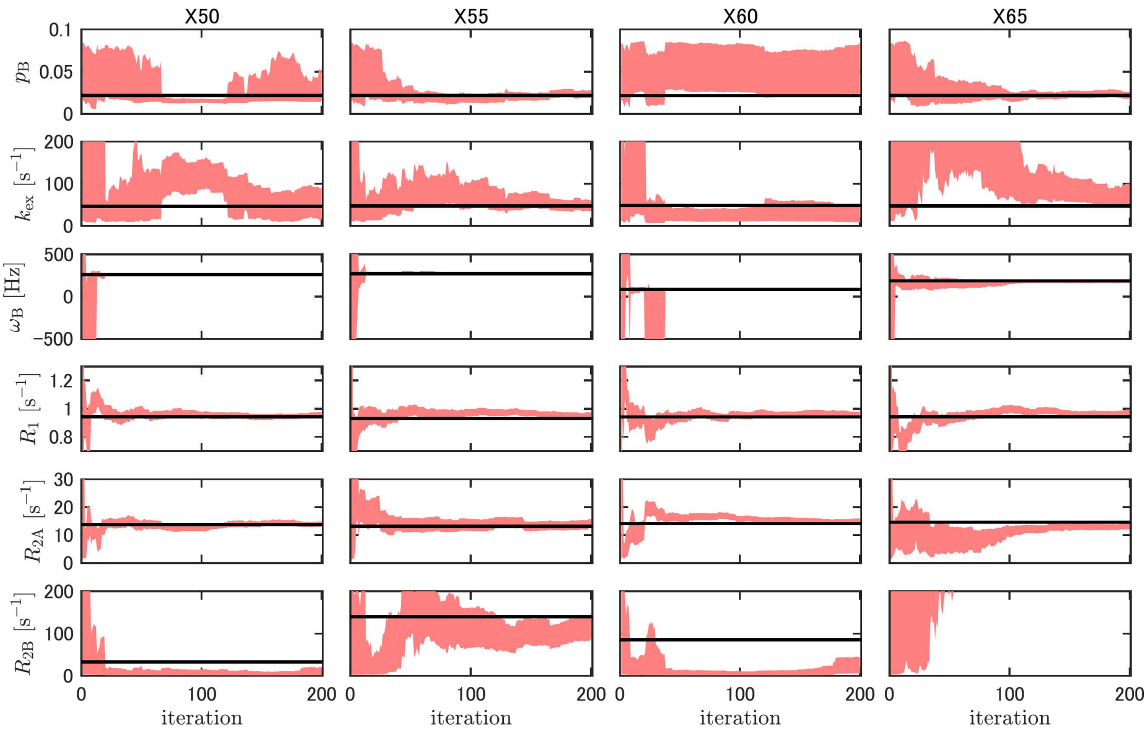

S13 Figure. The model-parameter estimation for the representative residues of the adaptive CEST simulation A3. Red areas represent 68.3 % credible intervals (CIs) for the estimated model parameters. Horizontal black lines represent the actual parameter values.
