# Supplementary material for: Autonomous adaptive optimization of NMR experimental conditions for precise inference of minor conformational states of proteins based on chemical exchange saturation transfer
Source: PLoS One. 2025 May 16;20(5):e0321692. doi: 10.1371/journal.pone.0321692 (PMC12083826; doi:10.1371/journal.pone.0321692)
Supplement: S14 Fig — (PDF) [file pone.0321692.s014.pdf]

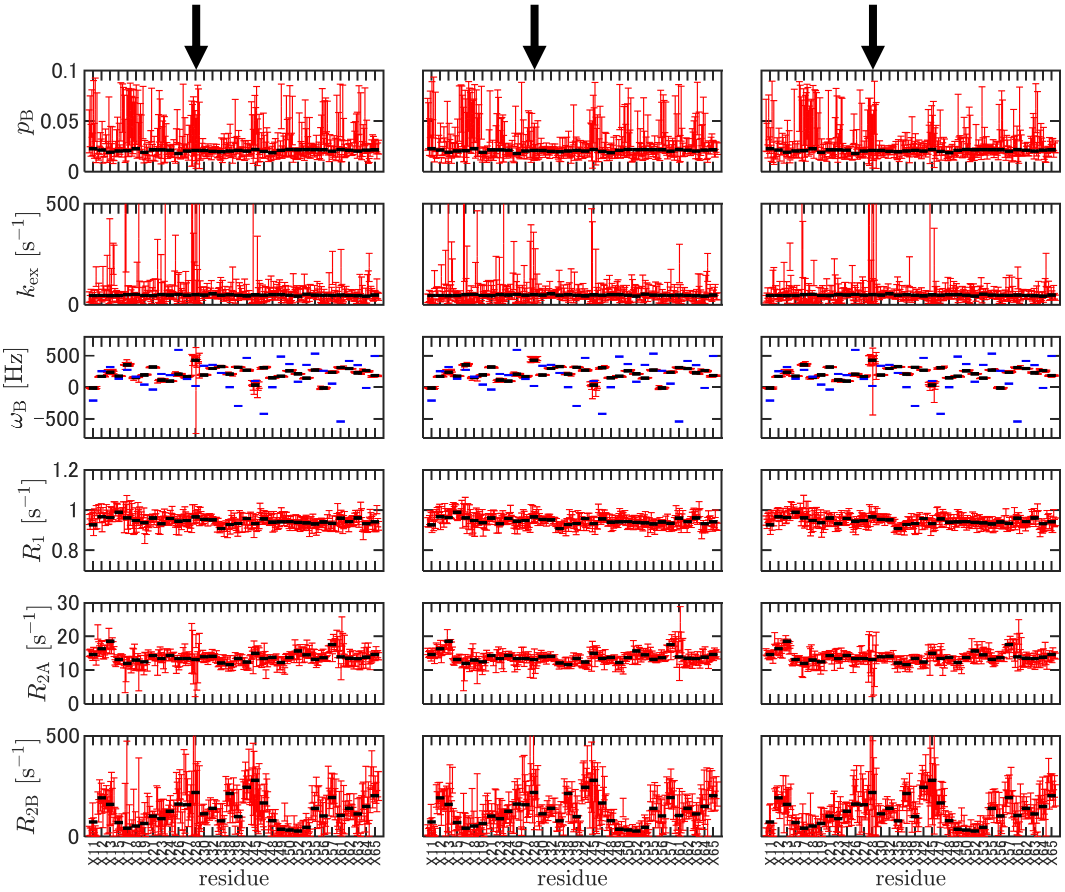

S14 Figure. The estimated model parameters with or without additional iterations for the simulation A3. (Left) Re-display of Fig 4a left. No additional iterations. (Middle) The estimated parameters after 240 iterations in total, consisting of 192 iterations of the original simulation A3 and the following additional 48 iterations using the different utility function, which is the mutual information of residue X28 only. (Right) The estimated parameters after 240 iterations by just extending 48 iterations to the simulation A3 using the same utility function which is the sum of the mutual information of all the residues. Residue X28 is indicated by arrows.
