# Supplementary material for: Autonomous adaptive optimization of NMR experimental conditions for precise inference of minor conformational states of proteins based on chemical exchange saturation transfer
Source: PLoS One. 2025 May 16;20(5):e0321692. doi: 10.1371/journal.pone.0321692 (PMC12083826; doi:10.1371/journal.pone.0321692)
Supplement: S15 Table — (PDF) [file pone.0321692.s015.pdf]

S15 Table. Detailed information for NMR measurements.

experiment type	$^1H/^{15}N$ HSQC	^{15}N CEST
manufacturer	Bruker BioSpin GmbH	
model	AVANCE III HD	
field strength	700.133 MHz	
probehead	TCI CryoProbe (CP TCI 700S4 H/C-N-D-05 Z)	
pulse program name	hsqcetf3gp	kas_hsqc_cest_etf3gpsitc2d
nuclei being measured	^1H (direct dimension)<br>^{15}N (indirect dimension)	
sample concentration	0.1 mM	
solvent in which the sample is dissolved	100 mM sodium acetate, 200 mM sodium chloride, 10% (v/v) D_2O, pH 5.7	
solvent signal suppression	pulsed field gradients	
chemical shift reference	The spectrum is indirectly referenced through the deuterium solvent lock signal according to the manufacturer's protocols.	
temperature	274.2 K	
